# Altered kynurenine pathway metabolites in a mouse model of human attention-deficit hyperactivity/autism spectrum disorders: A potential new biological diagnostic marker

**DOI:** 10.1038/s41598-019-49781-y

**Published:** 2019-09-12

**Authors:** Yuki Murakami, Yukio Imamura, Kuniaki Saito, Daisuke Sakai, Jun Motoyama

**Affiliations:** 1 Organization for Research Initiatives and Development, Doshisha University, Kyoto, 610-0394 Japan; 2 Department of Hygiene and Public Health, Kansai Medical University, Hirakata, 573-1010 Osaka Japan; 3 Department of Traumatology and Acute Critical Medicine, Osaka University Graduate School of Medicine, Suita, 565-0871 Osaka Japan; 4 Department of Disease Control and Prevention, Fujita Health University Graduate School of Health Sciences, Toyoake, 470-1192 Japan; 5 Division of General Education, Biology, Kanazawa Medical University, Kanazawa, 920-0293 Japan; 6 Laboratory of Development Neurobiology, Graduate School of Brain Science, Doshisha University, Kyoto, 610-0394 Japan

**Keywords:** Diagnostic markers, Neurodevelopmental disorders

## Abstract

Deleterious mutations in patchd1 domain containing 1 (*PTCHD1*) gene have been identified in patients with intellectual disability and/or autism spectrum disorder (ASD). To clarify the causal relationship between *Ptchd1* deficiency and behavioral defects relevant to neurodevelopmental disorders, we generated global *Ptchd1* knockout (KO) mice. *Ptchd1* KO mice displayed hyperlocomotion, increased impulsivity, and lower recognition memory, which resemble attention-deficit hyperactivity disorder (ADHD)-like behaviors. Acute or chronic treatment with atomoxetine ameliorated almost all behavioral deficits in *Pthcd1* KO mice. We next determined possible involvement of the kynurenine pathway (KP) metabolites in neurodevelopmental disorders in *Ptchd1* KO mice and assessed the potential of KP metabolites as biomarkers for ADHD and/or ASD. *Ptchd1* KO mice showed drastic changes in KP metabolite concentrations in the serum and the brain, indicating that the activated KP is associated with ADHD-like behaviors. Our findings indicate that *Ptchd1* KO mice can be used as an animal model of human ADHD and/or ASD, and KP metabolites are potential diagnostic biomarkers for neurodevelopmental disorders.

## Introduction

Attention deficit hyperactivity disorder (ADHD) and autism spectrum disorder (ASD) are two of the most common neurodevelopmental disorders with a prevalence of 1% in children aged 5–7 years. ADHD is characterized by inattention, hyperactivity, impulsivity, or a combination of these symptoms. Distinct symptoms of ASD include deficits in social communication and restrictive, repetitive and stereotyped patterns of behavior. Most children with ASD, including those with comorbid intellectual disability (ID), display impulsive, inattentive, and hyperkinetic symptoms. Approximately 40–70% of ASD children also fulfill the criteria for ADHD^1,2^. Likewise, 18–22% of ADHD children without ID display significant social communication impairment and lack of flexibility^3,4^, and 10% fulfill the criteria for ASD. Recent genetic studies have revealed a substantial overlap in risk genes in these two disorders^5–7^. In addition, both disorders are affected by several environmental risk factors, including prenatal immune challenge^8^ and maternal lifestyle factors, such as smoking, alcohol, and drug use, during the prenatal period^9,10^. Both disorders are typically diagnosed in childhood, but affected individuals frequently remain symptomatic even in adulthood^11^. Currently, the diagnosis of ADHD and ASD in children is made by experts using a combination of developmental history, collateral information, psychometrics, and observation of behavioral impairments according to the criteria of the Diagnostic and Statistical Manual of mental disorders, fifth edition (DSM-5)^12^. However, the diagnosis is often challenging because these diagnostic approaches are less objective and time consuming. Thus, an appropriate diagnosis needs to consider the applicability of the diagnostic approach. Compared with children with ADHD only, those with comorbid ADHD and ASD tend to be prescribed with more medications^13^, respond less to stimulants^14^, and may respond better to alternative agents, such as atomoxetine (ATX). Therefore, it is quite important to develop an objective medical test, such as a blood test, for the diagnosis of ADHD and ASD. Several studies from genomics to metabolomics, as well as those including measurements of monoamine (e.g., dopamine [DA], serotonin [5-HT], and noradrenaline [NA])^15^, hormone (e.g., cortisol and oxytocin)^16^, and neurotrophic factor (e.g., brain-derived neurotrophic factor [BDNF])^17^ levels in the plasma, have been performed to identify potentially predictive peripheral and genetic biomarkers for ADHD and ASD^18^. However, these studies are not conclusive, and further investigation is thus needed to develop optimal predictive biomarkers.

Mutations in the patched domain containing 1 gene (*PTCHD1*) on chromosome X are one of the most frequently identified ASD risk factors^19^. Comprehensive clinical analysis indicates that patients carrying *PTCHD1* deletions have a variable non-syndromic neurodevelopmental disorder with symptoms including attention deficit, hyperactivity, sleep abnormality, hypotonia, and learning disability^20^. Subsequent studies have confirmed the association of common and rare variants of *PTCHD1* gene with ASD and ID in several patient populations^21,22^. In addition, *Ptchd1* mutant mice show behavioral alterations, in particular, ADHD-like behaviors, such as hyperactivity, learning impairments, hyper-aggression, motor defects, and inhibitory avoidance and defects in contextual fear conditioning^23–25^. Moreover, *Ptchd1* mutant mice exhibit increased rearing in different environments, which represents a common feature of ASD and is considered a repetitive motor behavior classically used to reflect stereotypic behavior^24^. Therefore, *Ptchd1* mutant mice can be used as an animal model of ADHD and/or ASD in terms of construct validity, such as structural and functional impairments, and face validity, such as abnormal behaviors. Pharmacological boosting of small-conductance Ca^+^-activated potassium channels has been demonstrated to rescue ADHD-like behaviors in *Ptchd1* knockout (KO) mice^23^; however, to the best of our knowledge, no study has investigated whether therapeutic drugs for humans can reverse the abnormal behaviors observed in these mice.

In addition to genetic origin, bacterial or viral infections, exposure to environmental or dietary toxins, or experience of stressful situations may also cause ADHD and/or ASD^8–10^. These factors can directly affect the kynurenine pathway (KP), which is the main pathway involved in tryptophan (TRP) degradation (Supplemental Fig. S5). Infections induce an immune response that increases the production of several proinflammatory cytokines, including tumor necrosis factor (TNF)-α, interleukin (IL)-6, IL-1β, and interferon (IFN)-γ. These cytokines activate the first rate-limiting KP enzymes, including indoleamine-2,3-oxygenase1 (IDO1) and tryptophan-2,3-dioxygenase (TDO), as well as the second rate-limiting KP enzyme, kynurenine monooxygenase (KMO)^26^. Notably, several studies have demonstrated the association of ADHD and/or ASD with inflammatory mechanisms, as indicated by significantly high levels of numerous inflammatory cytokines in patients^27–32^. In addition, psychological stress increases synthesis and secretion of adrenal cortisol hormones, which affect the KP in the peripheral system and the brain^33^. The hypothalamus-pituitary-adrenal axis is dysregulated in children with ASD compared to healthy peers^34^, perhaps because of elevated stress and anxiety levels in ASD children^35^. Moreover, some natural products, such as brassinin and epacadostat, can inhibit IDO1 activity and reduce KP metabolites^36,37^. Furthermore, imbalance of KP metabolites, such as kynurenic acid (KYNA) and quinolinic acid (QUIN), is involved in several psychiatric disorders, including schizophrenia and depression, as well as ADHD and ASD^38–42^. Previous studies including ours have shown that endogenous kynurenine (KYN), KYNA, and anthranilic acid (AA) levels are markedly increased in mice with targeted KMO deletion, and offspring of KMO KO dams exhibit both depression-like^42^ and ASD-like behaviors^43^. Therefore, in this study examined the role of KP metabolites in *Ptchd1* KO mice to elucidate biological mechanisms underlying ADHD and/or ASD, and the findings would provide some insights into the development of new predictive biomarkers for these neurodevelopmental disorders.

In the present study, we generated global *Ptchd1* KO mice as an animal model of ADHD and/or ASD and examined whether this new mouse line has abnormal behavioral phenotypes observed in other *Ptchd1* mutant mouse lines. Subsequently, we evaluated the pharmacological effect of ATX, a non-stimulant drug available for ADHD and/or ASD patients, on the behavioral deficits of *Ptchd1* KO mice. Moreover, we measured the levels of KP metabolites in the serum and frontal cortex of *Ptchd1* KO mice to elucidate the involvement of the activated KP in ADHD pathophysiology and to assess the potential of KP metabolites as predictive peripheral biomarkers for neurodevelopmental disorders.

## Results

### Generation and behavioral abnormalities of *Ptchd1* KO mice

Previous studies employing *Ptchd1* KO mice have generated a conditional allele of *Ptchd1* by targeting exon 2. Because exon 2 encodes 3 out of the 12 transmembrane domains of Ptchd1 receptor, including a substantial portion of the sterol-sensing domain, its deletion generates a truncated, non-functional protein^23–25^. In contrast, a partial deletion encompassing the first exon was reported to disrupt *PTCHD1* in patients with ASD and/or ID^44^. Therefore, we generated a mouse model carrying exon 1 deletion to investigate the human model of neurodevelopmental disorders (Supplemental Fig. S1A). We confirmed the absence of full-length *Ptchd1* and deletion of the target locus using genomic PCR (Supplemental Fig. S1B).

The gross appearance was not obviously different between WT and *Ptchd1* KO littermates. Male *Ptchd1* KO mice were fertile, while female mice proceeded normally through gestation and parturition (data not shown).

**Figure 1:**
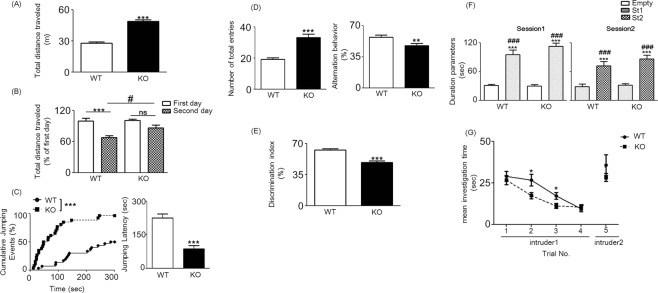
Behavioral abnormalities related to attention-deficit hyperactivity disorder in *Ptchd1* knockout (KO) mice. (**A**) Locomotor activity in a novel environment. Total distance traveled for 10 min was measured as an index of locomotor activity [Student’s *t-*test: ^***^p < 0.001 versus wild type (WT)]. (**B**) Habituation to a novel environment. The ration (percent) of total distance traveled on the second day to that traveled on the first day was significantly higher in *Ptchd1* KO than in WT mice during repeated trials, suggesting a decreased habituation of *Ptchd1* KO mice to the novel environment [two-way analysis of variance (ANOVA) with Bonferroni’s multiple comparison test: ^***^p < 0.001 versus WT on first day; ^#^*p* < 0.05 versus WT on second day]. (**C**) Impulsivity in the cliff avoidance test. The latency to jump off the cliff was measured. The cumulative curve of jumping events was generated by the Kaplan-Meier method (log-rank test with Bonferroni’s correction: ^***^p < 0.001 versus WT). The averages of the jumping latency are shown (Student’s *t*-test: ^***^p < 0.001 versus WT). (**D**) Spontaneous activity and working memory in the Y-maze test. Total arm entries and alternation behavior were measured during an 8-min session (Student’s *t*-test: ^**^p < 0.01 and ^***^p < 0.001 versus WT). (**E**) Objective recognition memory was measured in a novel object-based recognition test. Memory retention session was assessed 24 h after the training session. The discrimination index was calculated as shown in Methods (Student’s *t*-test: ^***^p < 0.001 versus WT). (**F**) Three-chamber social approach test. Dura*t*ion parameters are shown as investigation times (two-way ANOVA with Bonferroni’s multiple comparison test: ^***^p < 0.001 versus WT to Empty; ^###^*p* < 0.001 versus KO to Empty). No significant difference was found between groups. Empty, empty cage; St1, stranger 1; St2, stranger 2. (**G**) Five-trial social recognition memory test. The investigation time in repeated trials was significantly shorter in *Ptchd1* KO than in WT mice (Trial 2 and 3; two-way ANOVA with repeated measurements: ^*^p < 0.05 versus WT). Data are represented as mean ± standard error of the mean (SEM; n = 17–19).

To characterize neuronal function, we conducted several behavioral tests, including the novel open field test (OFT), cliff avoidance reaction, Y-Maze, novel object recognition test (NORT), three-chamber social approach, and five-trial social recognition memory tests. In the OFT, *Ptchd1* KO mice exhibited a significant increase in total distance travelled in the novel environment (Student’s *t*-test: t = 10.97, *p* < 0.0001; Fig. 1A) and a less decreased travel distance during repeated trials [two way ANOVA: F_second day (1, 68)_ = 17.77, *p* < 0.0001; F_*Ptchd1* KO (1, 68)_ = 5.61, *p* = 0.0208, F_second day × *Ptchd1* KO (1, 68)_ = 5.61, *p* = 0.0208; Fig. 1B], suggesting a difficulty in habituating to the new environment. The behavioral symptoms are similar to those in other ADHD model animals. However, a decrease in time spent in the center of the open field, which is an indicator of increased anxiety-like behavior^45^, was not observed in both *Ptchd1* KO and WT mice (Supplemental Fig. S2A: Student’s *t*-test, t = 0.394, *p* > 0.05). In the cliff avoidance test, the jumping latency was significantly shorter in *Ptchd1* KO mice than in WT mice (log-rank test: *p* < 0.0001; Student’s *t*-test: t = 5.93, *p* < 0.0001; Fig. 1C). Approximately half of the *Ptchd1* KO mice jumped down within 2 min. In the Y-maze test, *Ptchd1* KO mice showed higher spontaneous hyperactivity and altered working memory compared with their WT littermates, as indicated by the significantly reduced percentage of spontaneous alternation behaviors (Student’s *t*-test: t = 2.82, *p* = 0.0069; Fig. 1D). In the NORT, *Ptchd1* KO mice displayed a significantly lower performance than did their WT littermates (Student’s *t*-test: t = 6.34, *p* < 0.0001; Fig. 1E). However, we could not find any abnormal social behaviors in *Ptchd1* KO mice [two-way ANOVA: F_empty (1, 44)_ = 149.2, *p* < 0.0001; F_stranger 1 (1,44)_ = 0.38, *p* = 0.54; F_empty × stranger 1 (1, 44)_ = 1.11, *p* = 0.30; Fig. 1F], as assessed in the three-chamber social approach test. Moreover, in the five-trial social recognition memory test, *Ptchd1* KO mice did not show significant differences in the mean investigation time in the first and fourth trial with intruder 1 and the fifth trial with a new intruder [intruder 2; repeated measures ANOVA: F _*ptchd1* KO (1, 22)_ = 7.24, *p* = 0.0133; F _trials (1, 22)_ = 18.87, *p* < 0.0001; F _*Ptchd1* KO × trials (1, 22)_ = 1.58, *p* = 0.187; Fig. 1G]. However, *Ptchd1* KO mice became habituated faster than WT littermates and did not find the same intruder as interesting as the completely new intruder (Fig. 1G). Forced swim test (FST) was used to assess depression-like behavior. Notably, *Ptchd1* KO mice showed significantly reduced immobility time in the FST, probably because of hyperkinesia in these mice (Supplemental Fig. S2B).

### Acute and chronic ATX treatment improves the behavioral abnormalities of *Ptchd1* KO mice

**Figure 2:**
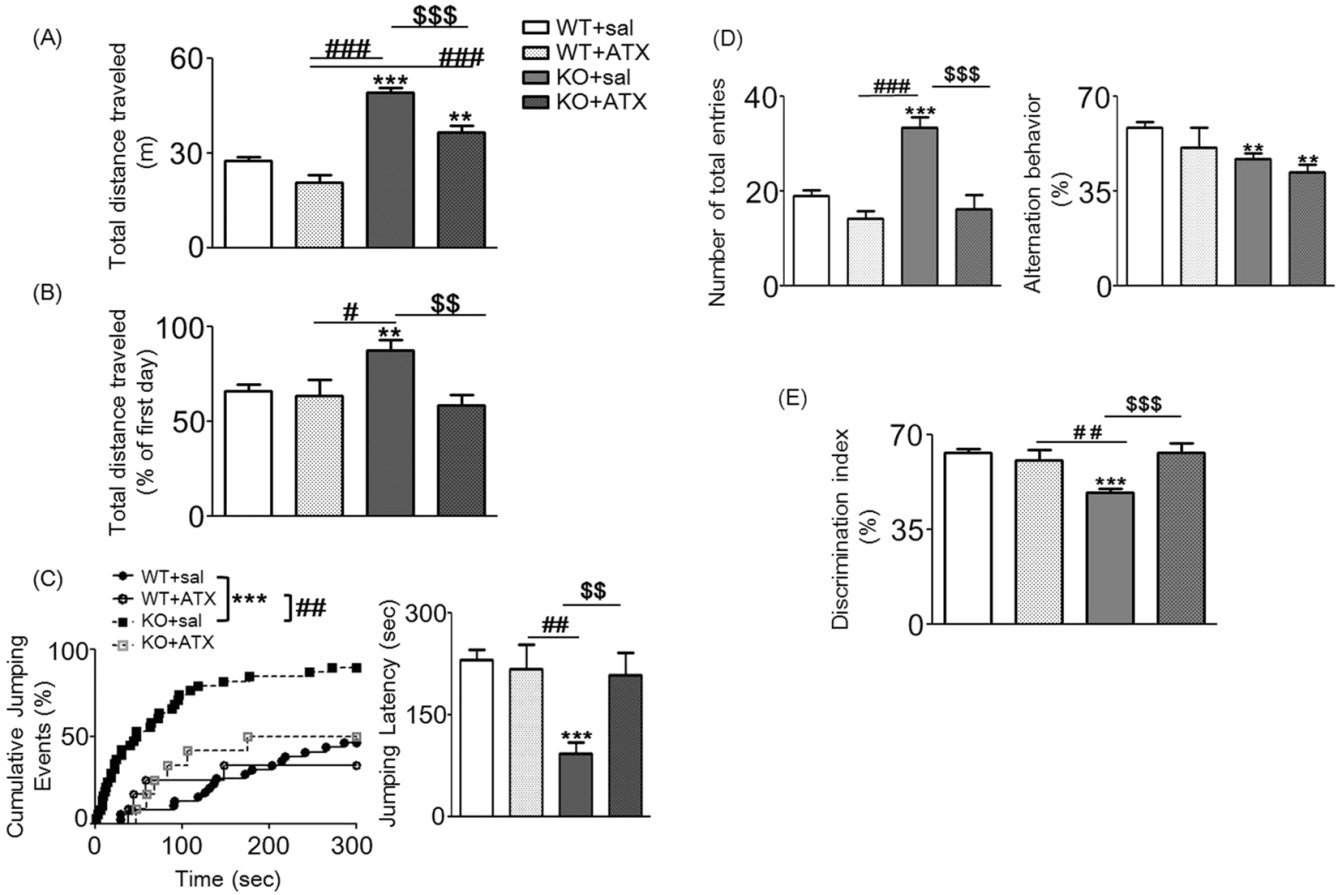
Acute atomoxetine (ATX) treatment ameliorates the behavioral abnormalities of *Ptchd1* knockout (KO) mice. ATX was administrated at a dose of 3.0 mg/kg to 8-week-old wild type (WT) and *Ptchd1* KO mice 30 min before the behavioral tests. (**A**) Total distance traveled in a novel environment on the first day [two-way analysis of variance (ANOVA) with Bonferroni’s multiple comparison test: ^***^p < 0.001 versus WT + sal; ^###^*p* < 0.001 versus WT + ATX; ^$$$^*p* < 0.001 versus KO + sal]. (**B**) Percentage of total distance traveled on the second day to that traveled on the first day, a measure of habituation to a novel environment (two-way ANOVA with Bonferroni’s multiple comparison test: ^*^p < 0.05 versus WT + sal; ^#^*p* < 0.05 versus WT + ATX; ^$^*p* < 0.05 versus KO + sal). (**C**) Impulsivity in the cliff avoidance test. The cumulative curve of the jumping events was generated by the Kaplan-Meier method (log-rank test with Bonferroni’s correction: ^***^p < 0.001 versus WT + sal; ^##^*p* < 0.01 versus WT + ATX). The average jumping latency is shown (two-way ANOVA with Bonferroni’s multiple comparison test: ^***^p < 0.001 versus WT + sal; ^##^*p* < 0.01 versus WT + ATX; ^$^*p* < 0.05 versus KO + sal). (**D**) Spontaneous activity and working memory in the Y-maze test. Total arm entries ^(^two-way ANOVA with Bonferroni’s multiple comparison test: ^***^p < 0.001 versus WT + sal; ^###^*p* < 0.001 versus WT + ATX; ^$$$^*p* < 0.001 versus KO + sal) and alternation behavior (two-way ANOVA with Bonferroni’s multiple comparison test: ^*^p < 0.05 and ^**^p < 0.01 versus WT + sal) were measured during an 8-min session. (**E**) Objective recognition memory was measured in the novel object-based recognition test. Memory retention session was assessed 24 h after the training session. The discrimination index was calculated as shown in Methods (two-way ANOVA with Bonferroni’s multiple comparison test: ^***^p < 0.001 versus WT + sal; ^#^*p* < 0.05 versus WT + ATX; ^$$$^*p* < 0.001 versus KO + sal). Data are shown as mean ± standard error of the mean (SEM; n = 10–28). Sal, saline.

Based on the above results, we validated that *Ptchd1* KO mice satisfied the condition of face validity as an animal model of ADHD. To assess whether the behavioral abnormalities of *Ptchd1* KO mice could be reversed by treatment with ATX, a medication for ADHD and/or ASD, we investigated the effect of acute ATX administration in *Ptchd1* KO mice. Compared with treatment with saline, acute treatment with ATX at a dose of 3 mg/kg significantly reduced the spontaneous locomotor activity of *Ptchd1* KO mice [two-way ANOVA: F _*Ptchd1* KO (1, 69)_ = 25.05, *p* < 0.0001; F _ATX (1, 69)_ = 110.25, *p* < 0.0001; F _*Ptchd1* KO × ATX (1, 69)_ = 1.77, *p* = 0.187; Fig. 2A]. However, acute ATX treatment could not completely reverse the abnormal hyperlocomotion observed in *Ptchd1* KO mice. ATX reduced total distance travelled in the OFT in WT mice. Therefore, it is suspicious that treatment with ATX can reverse the hyperlocomotion of *Ptchd1* KO mice in a novel environment. In addition, acute ATX treatment significantly improved the reduced habituation of *Ptchd1* KO mice to the novel environment [two-way ANOVA: F _*Ptchd1* KO (1, 66)_ = 7.09, *p* = 0.01; F _ATX (1, 66)_ = 1.98, *p* = 0.164; F _*Ptchd1* KO × ATX (1, 66)_ = 5.24, *p* = 0.025; Fig. 2B], the shortened jumping latency in the cliff avoidance test [two-way ANOVA: F _*Ptchd1* KO (1, 92)_ = 2.89, *p* = 0.092; F _ATX (1, 92)_ = 8.38, *p* = 0.005; F _*Ptchd1* KO × ATX (1, 92)_ = 6.29, *p* = 0.014; Fig. 2C], spontaneous hyperactivity in the Y maze [two-way ANOVA: F _*Ptchd1* KO (1, 69)_ = 24.09, *p* < 0.0001; F _ATX (1, 69)_ = 13.13, *p* = 0.0006; F _*Ptchd1* KO × ATX (1, 69)_ = 7.52, *p* = 0.008; Fig. 2D], and the lower discrimination index in the NORT [two-way ANOVA: F _*Ptchd1* KO (1, 77)_ = 4.85, *p* = 0.031; F _ATX (1, 77)_ = 5.96, *p* = 0.017; F _*Ptchd1* KO × ATX (1, 77)_ = 11.85, *p* = 0.0009; Fig. 2E]. However, treatment with ATX was ineffective in recovering working memory impairments in the Y maze test [two-way ANOVA: F _*Ptchd1* KO (1, 62)_ = 3.31, *p* = 0.074; F _ATX (1, 62)_ = 9.04, *p* = 0.004; F _*Pthcd1* KO × ATX (1, 62)_ = 0.13, *p* = 0.723; Fig. 2D].

**Figure 3:**
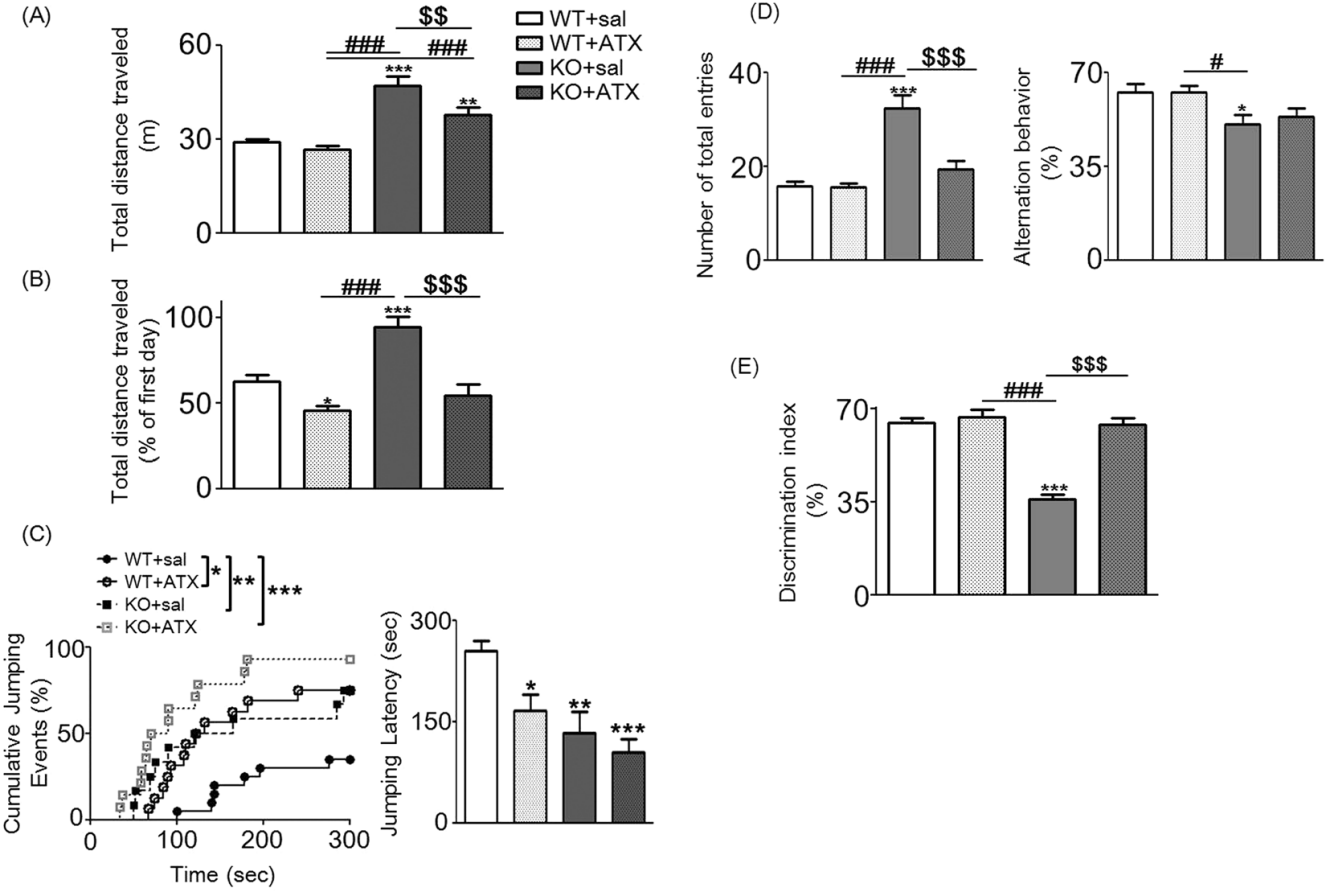
Chronic treatment with atomoxetine (ATX) restores behavioral abnormalities in *Ptchd1* knockout (KO) mice. ATX was administrated once per day for two weeks at a dose of 3.0 mg/kg in 6-week-old *Ptchd1* KO mice. Behavior tests were performed 30 min after injection in mice at the age of 8 weeks. (**A**) Total distance traveled in a novel environment was used as a measure of locomotor activity [two-way analysis of variance (ANOVA) with Bonferroni’s multiple comparison test: ^**^p < 0.01 and ^***^p < 0.001 versus WT + sal; ^###^*p* < 0.001 versus WT + ATX; ^$$^*p* < 0.01 versus KO + sal]. (**B**) Ratio (in percent) of total distance traveled on the second day to that traveled on the first day, a measure of habituation to a novel environment (two-way ANOVA with Bonferroni’s multiple comparison test: ^*^p < 0.05 and ^**^p < 0.01 versus WT + sal; ^###^*p* < 0.001 versus WT + ATX; ^$$$^*p* < 0.001 versus KO + sal). (**C**) Impulsivity in the cliff avoidance test. The cumulative curve of jumping events was generated by the Kaplan-Meier method (log-rank test with Bonferroni’s correction: ^*^p < 0.05, ^**^p < 0.01, ^***^p < 0.001 versus WT + sal). The average jumping latency is shown *(*two-way ANOVA with Bonferroni’s multiple comparison test: ^*^p < 0.05, ^**^p < 0.01, ^***^p < 0.001 versus WT + sal). (**D**) Spontaneous activity and working memory in the Y-maze test. Total arm entries (two-way ANOVA with Bonferroni’s multiple comparison test: ^***^p < 0.001 versus WT + sal; ^###^*p* < 0.001 versus WT + ATX; ^$$$^*p* < 0.001 versus KO + sal) and alternation behavior (two-way ANOVA with Bonferroni’s multiple comparison test: ^*^p < 0.05 versus WT + sal^; #^*p* < 0.05 versus WT + ATX) were measured during an 8-min session. (**E**) Objective recognition memory was measured in a novel object-based recognition test. Memory retention session was assessed 24 h after the training session. The discrimination index was calculated as described in Methods (two-way ANOVA with Bonferroni’s multiple comparison test: ^***^p < 0.001 versus WT + sal; ^###^*p* < 0.001 versus WT + ATX; ^$$$^*p* < 0.001 versus KO + sal). Data are shown as mean ± standard error of the mean (SEM; n = 12–20). Sal, saline.

ATX has a negligible risk of abuse and is considered particularly useful for children with ADHD and/or ASD, and its benefits in patients can be observed between 2–8 weeks after initial treatment^46^. Therefore, *Ptchd1* KO mice were administered ATX for 2 weeks before behavioral testing to investigate the effect of chronic ATX treatment. Notably, chronic and acute treatment had similar beneficial effects (Fig. 3A–E, Table 1). Chronic ATX treatment significantly improved the decreased habituation of *Ptchd1* KO mice to the novel environment [two-way ANOVA: F _*Ptchd1* KO (1, 46)_ = 19.83, *p* < 0.0001; F _ATX (1, 46)_ = 18.34, *p* < 0.0001; F _*Ptchd1* KO × ATX (1, 46)_ = 1.28, *p* = 0.264; Fig. 3B], spontaneous hyperactivity in the Y maze [two-way ANOVA: F _*Ptchd1* KO (1, 56)_ = 18.25, *p* < 0.0001; F _ATX (1, 56)_ = 42.80, *p* < 0.0001; F _*Ptchd1* KO × ATX (1, 56)_ = 17.18, *p* = 0.0001; Fig. 3D], and the lower discrimination index in the NORT [two-way ANOVA: F _*Ptchd1* KO (1, 55)_ = 41.31, *p* < 0.0001; F _ATX (1, 55)_ = 46.01, *p* < 0.0001; F _*Ptchd1* KO × ATX (1,55)_ = 29.83, *p* < 0.0001; Fig. 3E]. However, the chronic treatment could not completely reverse hyperlocomotion of *Ptchd1* KO mice [two-way ANOVA: F _*Ptchd1* KO (1, 54)_ = 11.55, *p* = 0.001; F _ATX (1, 54)_ = 72.28, *p* < 0.0001; F _*Ptchd1* KO × ATX (1, 54)_ = 3.74, *p* = 0.058; Fig. 3A] or lower working memory in the Y-maze test [two-way ANOVA: F _*Ptchd1* KO (1, 54)_ = 0.21, *p* = 0.645; F _ATX (1, 54)_ = 12.32, *p* = 0.0009; F _*Ptchd1* KO × ATX (1, 54)_ = 0.21, *p* = 0.652; Fig. 3D]. In addition, this long-term treatment exacerbated the shortened jumping latency in the cliff avoidance test [two-way ANOVA: F _*Ptchd1* KO (1, 57)_ = 10.75, *p* = 0.002; F _ATX (1, 57)_ = 11.71, *p* = 0.001; F _*Ptchd1* KO × ATX (1, 57)_ = 0.42, *p* = 0.518; Fig. 3C]. Both acute and chronic ATX treatment did not affect the social behavior of *Ptchd1* KO mice (Supplemental Fig. S3). However, chronic ATX treatment significantly reduced the time spent in close proximity to the unfamiliar mouse in WT mice compared with other mice (Supplemental Fig. S3C). In addition, ATX treatment in WT mice significantly reduced the mean investigation time of both familiar (intruder 1) and unfamiliar (intruder 2) mice during repeated trials (Supplement Fig. S3B,D).

**Table 1:** Effects of acute and chronic treatment with atomoxetine (ATX) on the abnormal behaviors of *Ptchd1* knockout mice.

Behavior tests	ATX (−)	ATX (+)	
		Acute	Chronic
Spontaneous locomotor activity in a novel environment (OFT day1)	**(+)**	Improved?	Improved?
Habituation to a novel environment (Repeated exposure, OFT day2)	**(+)**	Improved	Improved
Impulsivity (Cliff avoidance test)	**(+)**	Improved	Aggravated
Spontaneous activity in Y maze	**(+)**	Improved	Improved
Working memory (Y maze)	**(+)**	Not improved	Not improved
Novel object recognition memory	**(+)**	Improved	Improved
Three-chamber social approach	(−)	No effect	No effect
Social recognition memory	(−)**/?**	No effect	No effect

**(+)**: significantly impaired compared with wild type mice; (−): not impaired compared with wild type mice. OFT, open-field test.

Psychostimulants, such as methylphenidate (MPH), are well-studied medications used to treat ADHD alone. Mice were administered MPH acutely before the tests to assess whether the behavioral abnormalities in *Ptchd1* KO mice can be ameliorated by treatment with psychostimulants. However, we could not find any improvement of the behavioral abnormalities after acute MPH treatment in *Ptchd1* KO mice (Supplemental Fig. S4).

### Remarkably altered KP metabolite concentrations in the serum and the brain in *Ptchd1* KO mice

**Figure 4:**
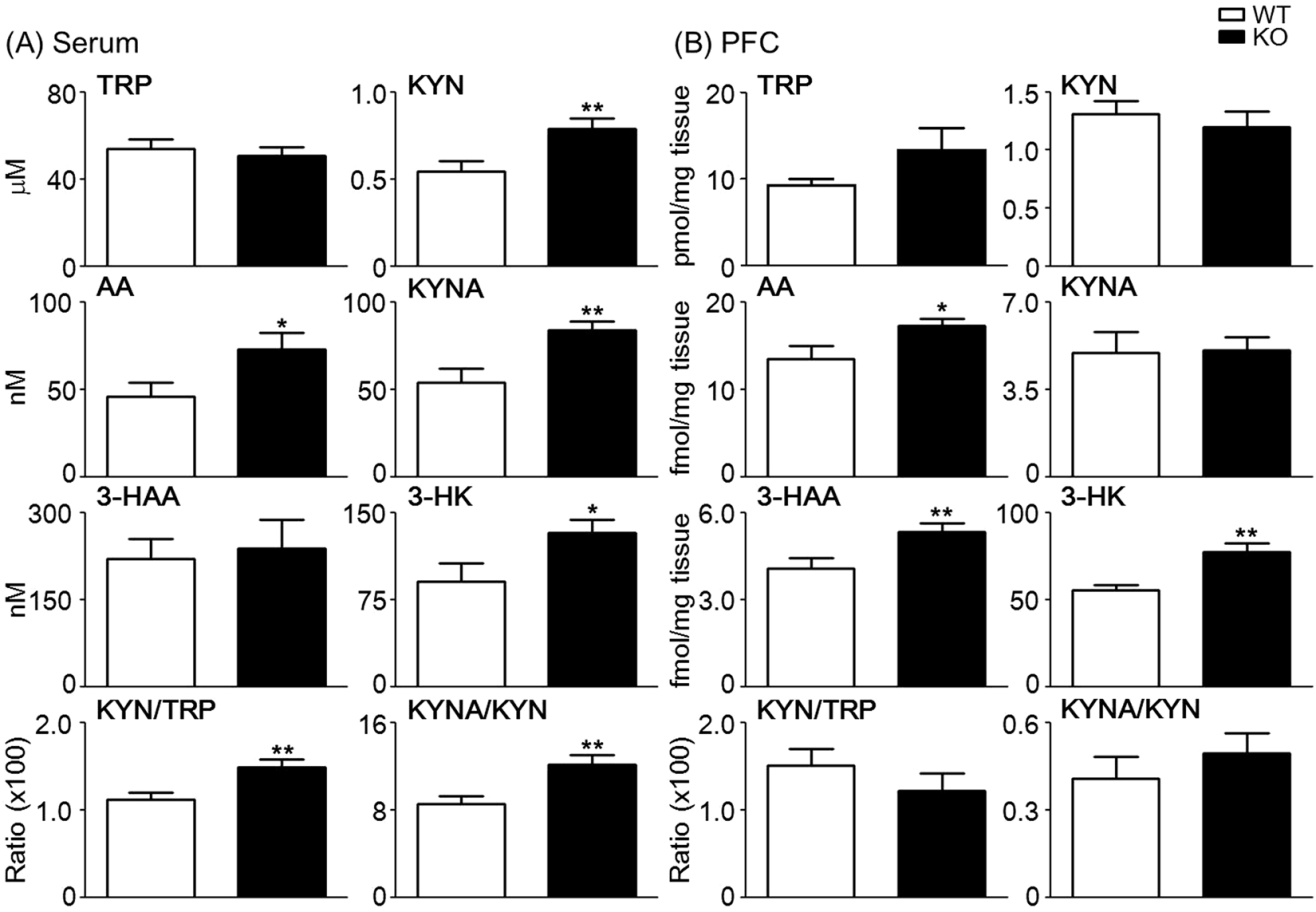
Remarkable changes in kynurenine pathway (KP) metabolite concentrations in the serum and brain of *Ptchd1* knockout (KO) mice. KP metabolite concentrations were determined in the serum (**A**) and the frontal cortex (**B**) of mice at 11 weeks of age. Open and closed bars correspond to wild-type (WT) mice and *Ptchd1* KO mice, respectively. Each column represents mean ± standard error of the mean (SEM; n = 13–15). Statistical analysis was conducted using Student’s *t*-test: ^*^p < 0.05, ^**^p < 0.01 versus WT mice.

To evaluate whether KP metabolites can be used as peripheral diagnostic biomarkers for ADHD and/ASD, we measured their levels in the serum and frontal cortex of *Ptchd1* KO mice. First, changes of KP metabolites in the serum in mice of different ages (6, 8, 11, 12, and 14 weeks of age) were measured (Supplemental Fig. S6). The concentrations of KYN and 3-HK were higher in the serum of *Ptchd1* KO mice than in that of WT mice at 6–11 weeks of age. We also found the significant difference in KP metabolites in the serum between 11-week-old *Ptchd1* KO and WT mice. The serum levels of KYN and AA were significantly higher (by 45.0 and 57.7%, respectively) in *Ptchd1* KO mice than in WT mice (KYN: WT versus KO, 0.54 ± 0.06 versus 0.79 ± 0.06 μM; t = 2.94, *p* = 0.007, Student’s *t*-test; AA: WT versus KO, 46.1 ± 7.6 versus 72.6 ± 9.6 nM; t = 2.17, *p* = 0.040, Student’s *t*-test; Fig. 4A). Significant increases in KYNA (WT versus KO, 53.6 ± 8.0 versus 83.8 ± 4.8 nM; t = 3.22, *p* = 0.003, Student’s *t-*test) and 3-hydroxykynunenine (3-HK; WT versus KO, 90.2 ± 16.1 versus 132.8 ± 11.3 nM; t = 2.17, *p* = 0.039, Student’s *t*-test) levels were found in KO mice, but no changes in TRP (WT versus KO, 53.9 ± 4.2 versus 50.8 ± 3.7 μM) and 3-hydroxyanthranilic acid (3-HAA; WT versus KO, 220.2 ± 34.0 versus 237.7 ± 49.4 nM) concentrations were observed in KO mice. The KYN/TRP ratio (reflecting IDO1 activity) (Student’s *t*-test: t = 3.03, *p* = 0.005) and the KYNA/KYN ratio [reflecting KYN aminotransferase (KAT) II activity] (Student’s *t* test: t = 3.03, *p* = 0.005) were significantly higher in KO mice than in WT mice.

In the frontal cortex, *Ptchd1* KO mice showed significantly higher levels of AA, 3-HAA, and 3-HK than did WT littermates (AA: WT versus KO, 13.5 ± 1.50 versus 17.3 ± 0.76 fmol/mg tissue; 28.5% increase; t = 2.47, *p* = 0.02, Student’s *t*-test; 3-HAA: WT versus KO, 4.06 ± 0.37 versus 5.35 ± 0.27 fmol/mg tissue; 31.5% increase; t = 2.86, *p* = 0.009, Student’s *t*-test; 3-HK: WT versus KO, 55.2 ± 3.0 versus 77.2 ± 5.2 fmol/mg tissue; 40.0% increase; t = 3.29, *p* = 0.003, Student’s *t*-test; Fig. 4B). There were a slight increase in TRP levels and a slight decrease in KYN levels, but no changes in KYNA levels. In addition, no significant changes in both KYN/TRP and KYNA/KYN ratios were found.

### Changes in KP metabolite concentrations in the serum and brain following ATX treatment

**Figure 5:**
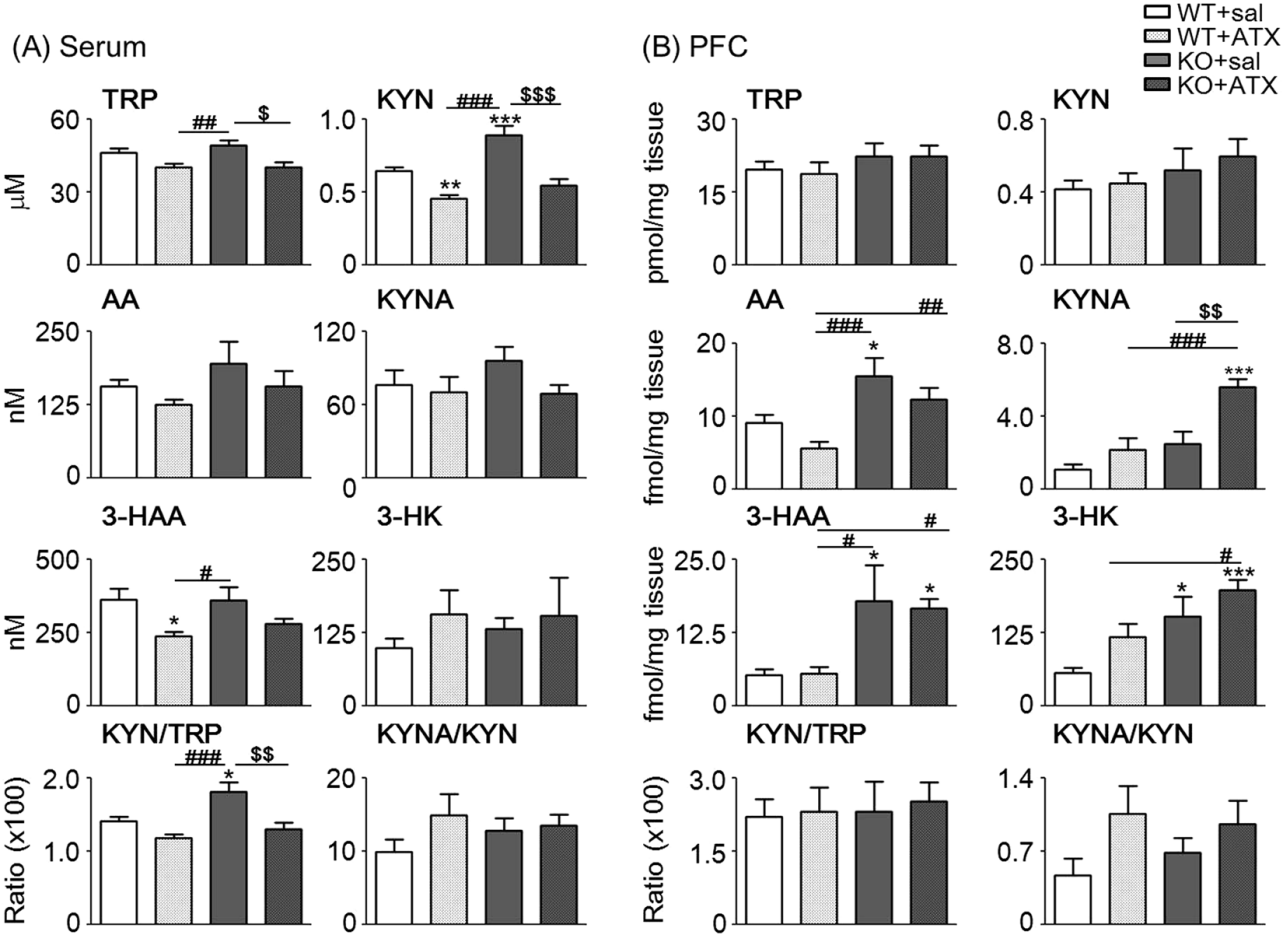
Effect of acute atomoxetine (ATX) treatment on kynurenine pathway (KP) metabolite concentrations in the serum and brain of *Ptchd1* knockout (KO) mice. KP metabolite concentrations were determined in the serum (**A**) and frontal cortex (**B**) of mice at 11 weeks of age 30 min after ATX treatment [two-way analysis of variance (ANOVA) with Bonferroni’s multiple comparison test: ^*^, *p* < 0.05, ^**^p < 0.01 and ^***^p < 0.001 versus WT + sal; ^#^*p* < 0.05, ^##^*p* < 0.01 and ^###^*p* < 0.001 versus WT + ATX; ^$^*p* < 0.05, ^$$^*p* < 0.01 and ^$$$^*p* < 0.001 versus KO + sal]. Data are shown as mean ± standard error of the mean (SEM; n = 8–16). Sal, saline.

To assess whether KP metabolites are implicated in pathological conditions of ADHD/ASD, we measured concentrations of KP metabolites following acute ATX treatment in *Ptchd1* KO and WT mice at 11 weeks of age. The serum level of KYN was significantly higher in *Ptchd1* KO mice than in WT mice, and ATX treatment significantly decreased the serum level of KYN in both mice [Saline versus ATX: 0.64 ± 0.02 versus 0.46 ± 0.02 μM in WT mice and 0.89 ± 0.06 versus 0.54 ± 0.05 μM in KO mice; two-way ANOVA: F _*Ptchd1* KO (1, 44)_ = 20.67, *p* < 0.0001; F _ATX (1, 44)_ = 4.86, *p* = 0.033; F _*Ptchd1* KO × ATX (1, 44)_ = 53.87, *p* < 0.0001; Fig. 5A]. The level of TRP in the serum also significantly decrease after ATX treatment in WT and *Ptchd1* KO mice [Saline versus ATX: 46.2 ± 1.6 versus 40.0 ± 1.7 μM in WT mice and 49.2 ± 1.9 versus 40.0 ± 2.1 μM in KO mice; two-way ANOVA: F _*Ptchd1* KO (1, 48)_ = 0.67, *p* = 0.416; F _ATX (1, 48)_ = 17.6, *p* = 0.0001; F _*Ptchd1* KO × ATX (1, 48)_ = 0.69, *p* = 0.412; Fig. 5A]. There were slight decreases in AA, KYNA, and 3-HAA levels following ATX treatment, but no changes in 3-HK levels, in *Ptchd1* KO mice. The KYN/TRP ratio (reflecting IDO1 activity) was significantly higher in *Ptchd1* KO than in WT mice but significantly decreased in *Ptchd1* KO mice after ATX treatment [Saline versus ATX: 1.40 ± 0.06 versus 1.17 ± 0.06 in WT mice and 1.81 ± 0.13 versus 1.29 ± 0.97 in KO mice; two-way ANOVA: F _*Ptchd1* KO (1, 42)_ = 10.4, *p* = 0.003; F _ATX (1, 42)_ = 20.9, *p* < 0.0001; F _*Ptchd1* KO × ATX (1, 42)_ = 2.97, *p* = 0.092; Fig. 5A]. In addition, the KYNA/KYN ratio (reflecting KAT II activity) did not change.

In the frontal cortex, only the level of KYNA showed significant changes after ATX treatment in *Ptchd1* KO mice [Saline versus ATX: 1.08 ± 0.28 versus 2.15 ± 0.65 fmol/mg tissue in WT mice and 2.48 ± 0.67 versus 5.58 ± 0.43 fmol/mg tissue in KO mice; two-way ANOVA: F _*Ptchd1* KO (1, 44)_ = 8.11, *p* = 0.0067; F _ATX (1, 44)_ = 5.69, *p* = 0.0214; F _*Ptchd1* KO × ATX (1, 44)_ = 3.74, *p* = 0.0596; Fig. 5B]. There were a slight decrease in the AA level and a slight increase in the 3-HK level following ATX treatment in both *Ptchd1* KO and WT mice. However, no change in the 3-HAA level was found. The KYN/TRP ratio (reflecting IDO1 activity) did not change, whereas the KYNA/KYN ratio (reflecting KAT II activity) slightly increased after ATX treatment.

## Discussion

ASD patients often exhibit comorbid conditions, including hyperactivity/inattention, aggression, anxiety, cognitive disability, and sleep disorders. Currently, the existence of hyperactivity/inattention in ASD patients is diagnosed as ASD comorbid with ADHD. Moreover, comprehensive clinical analysis of patients with *PTCHD1* mutations has revealed several symptoms, including hyperactivity/inattention and autistic symptoms^20^. Consistent with these clinical data, our new line of *Ptchd1* KO mice, in which the region containing exon 1 was deleted, showed hyperactivity, impaired working memory, and cognitive dysfunction, further confirming the phenotypes previously described in different lines of *Ptchd1*-deficient mice^23–25^. Moreover, our *Ptchd1* KO mice had increased impulsivity and decreased habituation to a novel environment, reminiscent of the clinical features of children with ADHD and/or ASD.

Core symptoms of ASD include impairments in social behavior and communication, as well as stereotypic behavior. Several mouse models of ASD exhibit impaired social behavior, measured by the lack of preference for a mouse over an object in the three-chamber social approach test^47–49^. However, we found normal sociability in *Ptchd1* KO mice, and the findings are consistent with those in previous studies using another line of *Ptchd1* KO mice^23^. Because patients with ASD often avoid unfamiliar social partners and show diminished interest in novelty, impairments in social novelty preference in the three-chamber social approach test (i.e., preference for a novel mouse over a familiar mouse) are used as indicators of lower social recognition in some ASD mouse models^50–52^. In this study, *Ptchd1* KO mice showed normal social novelty preference in the three-chamber approach test and five-trial social recognition memory test. Some ASD mouse models lacking ASD-related genes also present normal sociability and diminished social novelty preferences^53–55^. It has been suggested that the lack of sociability for unfamiliar targets is not always necessary for the diagnosis of ASD and might represent a symptom of individuals with ASD^56^. In the five-trial social recognition memory test with repeated trials, investigation time was shorter in *Ptchd1* KO than in WT mice. These data indicate that *Ptchd1* KO mice exhibit impaired social behavior and/or social recognition memory, although further studies are needed to confirm the findings. Taken together, *Ptchd1* KO mice can be considered a mouse model of comorbid ADHD and ASD based on the construct validity regarding structural and functional impairments and the face validity regarding abnormal behaviors.

To evaluate the predictive validity of *Ptchd1* KO mice, we studied the effect of a therapeutic medication for ADHD and ASD on the behavioral impairment of *Ptchd1* KO mice. In the clinical setting, psychostimulants are the most widely studied medications used to treat ADHD alone. However, conflicting findings have been reported regarding the efficacy and safety of these drugs, such as MPH, for ASD comorbid with ADHD. Moreover, MPH has significant negative side effects and limited therapeutic benefits, particularly for treating comorbid ADHD/ASD. Therefore, other non-stimulant medications have been investigated for comorbid ADHD and ASD symptoms. ATX, a non-stimulant medication, which is a selective NA reuptake inhibitor, can increase DA levels and has better tolerability than stimulant medications in individuals with comorbid ADHD and ASD. ATX can increase the concentrations of NA within the synapses by blocking NA transporters. Generally, ATX does not increase synaptic DA because DA transporters are not blocked by ATX. However, ATX can increase DA and NA particularly in the synaptic cleft of the prefrontal cortex (PFC). In the PFC, NA transporters play a central role in the reuptake of released DA and are highly expressed compared with DA transporters. Therefore, we investigated the effect of ATX administrations on our new line of *Ptchd1* KO mice.

Acute treatment with ATX improved the shortened jumping latency in the cliff avoidance test, the spontaneous activity in the Y maze test, and the novel object recognition memory, but not the impaired short-term working memory. Although ATX treatment reduced hyperlocomotion in a novel environment, it could not completely restore it back to normal levels (Fig. 2). Moreover, ATX did not affect social behavior (Supplemental Fig. S3A.B). ATX treatment slightly ameliorated hyperactivity in *Ptchd1* KO mice. This finding suggests that ATX may increase DA levels in the PFC, thus resulting in the improvement of hyperactivity. Notably, although MPH increased synaptic DA levels in the PFC, hyperactivity in *Ptchd1* KO mice was not ameliorated by MPH treatment. Several investigations have shown that MPH has no effect on the clearance of extracellular DA and total extracellular DA levels in subcortical areas involved in the regulation of locomotion, and DA and other neurotransmitters, such as 5-HT and NA, might be involved in hyperactivity in ADHD^57–60^. Acute ATX and MPH treatment did not increase the PFC levels of 5-HT in mice^60^; therefore, hyperkinesia might not be completely recovered back to the normal levels in WT mice. Further, we found that ATX improved impulsivity in *Ptchd1* KO mice, indicating that blocking of NA transporters may play a role in the effect. However, MPH did not improve the shortened latency despite its inhibitory effect on NA transporters.

MPH increases synaptic DA levels in the nucleus accumbens (NAC), striatum, and PFC by blocking DA transporters, but ATX does not increase synaptic DA in the NAC and striatum^61^. The different roles of MPH and ATX in dopaminergic transmission might explain their different effects on the pathological conditions of *Ptchd1* KO mice. Furthermore, numerous studies in ADHD patients have found that hypodopaminergia in the PFC is related to hyperactivity^62,63^. Notably, *Ptchd1* KO mice displayed a decreased recognition index in the NORT, which evaluates short-term working memory and visual attention. Because *Ptchd1* KO mice also exhibited impairments in short-term working memory in the Y-maze test, the reduced recognition index in *Ptchd1* KO mice may be related to both short-term memory deficits and visual attention deficits. Moreover, the reduced recognition index was recovered by treatment with ATX, but not MPH, suggesting that the attention deficits and hyperactivities may be regulated by different systems. Different mechanisms underlying the regulation of locomotion and cognition might be responsible for different subtypes of ADHD with hyperactive or inattentive components. However, further investigations into extracellular neurotransmitter levels following the treatment with ATX or MPH are needed to better understand the pathological condition in *Ptchd1* KO mice.

In contrast, chronic treatment with ATX did not show any additional benefits compared with acute treatment, and the shortened jumping latency became worse after chronic treatment with ATX (Fig. 3). Approximately half of ASD patients are classified as non-responders, and ATX’s efficacy may vary with impairment levels, including cognitive impairment levels and overall ASD symptom severities^64^. Sun *et al*. found that chronic treatment with ATX decreased the number of impulsive choices, i.e., the selection of small immediate rewards over large delayed rewards, but did not affect premature responding, i.e., impulsive actions, in rats in the five-choice reaction time task^65^. Although ATX could not reverse all abnormal behaviors in our model mice, our observations indicate that *Ptchd1* deficiency leads to structural and functional brain impairments reminiscent of the clinical features of comorbid ADHD and ASD. Therefore, *Ptchd1* KO mice show good predictive validity as an animal model for comorbid ADHD and ASD.

Evidence for altered KP metabolism in the pathophysiology of neurodevelopmental disorders, including ASD and ADHD, has been very limited to date. In this study, we found that the levels of KYN, AA, KYNA, and 3-HK and the KYN/TRP and KYNA/KYN ratios were significantly increased in the serum of adult *Ptchd1* KO mice (Fig. 4A). The TPR breakdown index (KYN/TRP ratio) was significantly higher in *Ptchd1* KO mice, and the findings are consistent with those in patients with ADHD and ASD. Moreover, significantly altered concentrations of KYN after ATX treatment correlated with behavioral improvements in *Ptchd1* KO mice (Fig. 5A), suggesting that altered KP metabolites, particularly a high level of KYN, might be related to the pathological conditions of ADHD/ASD. Our findings also suggest that KP metabolism is activated in the periphery, and that activated KP metabolism increases the levels of KYN, which can cross the blood brain barrier and generate more neuroactive compounds affecting neuronal function or brain neurodevelopment. Imbalances in TRP metabolism^66^ and a lower serum levels of 3-HK^30^ have been observed in children with ADHD, and children with ADHD have higher levels of cytokines, including IL-6 and IFN-γ, which are known to activate KP and correlate with clinical parameters of attention, impulsivity, and hyperactivity^31^. In addition, shorter pregnancy durations and lower birth weights are linked to symptom severities and increased 3-HK and IFN-γ levels in children with ADHD^32^. Moreover, ADHD children with or without comorbidities show significantly reduced AA and KYNA serum levels and increased TRP and KYN levels^67^. In contrast, ADHD is associated with lower concentrations of TRP, KYNA, and 3-HAA in the serum in adults^68^, and mean serum levels of TRP and KYNA and KYNA/KYN ratio are significantly lower in patients with ASD^69^.

The levels of AA, 3-HAA, and 3-HK in the frontal cortex significantly increased in *Ptchd1* KO mice (Fig. 4B). In this study, we could not measure the amount of QUIN, an N-methyl-D-aspartate receptor agonist that can cause neuronal toxicity in the brain^70,71^. However, our data indicate that downstream KP metabolites are catabolized to QUIN in the frontal cortex. Notably, only the concentration of KYNA was significantly changed in the frontal cortex of *Ptchd1* KO mice after ATX treatment (Fig. 5B). Very few studies have investigated the direct association between ADHD and increased QUIN levels, although several studies have shown that KP metabolite imbalances during prenatal neurodevelopment induces cognitive dysfunction, decreased social behaviors, and learning impairments^72–74^. In addition, Lim *et al*. found alternations in the KP, such as increased production of the downstream metabolite QUIN that enhances glutamatergic neurotransmission, in children with ASD^39^. These data suggest that QUIN could play a key role in the pathophysiology of these neurodevelopmental disorders. Several studies have indicated that even relatively moderate elevations in extracellular KYNA levels in the brain are functionally significant. KYNA is a broad-spectrum antagonist of ionotropic amino acid receptors. Especially, KYNA is a potent antagonist of α7 nicotinic acetylcholine (α7nACh) receptor^75,76^, and elevated extracellular KYNA in the striatum and PFC results in a marked reduction of extracellular glutamate levels^77,78^ and affects DA levels in the striatum^78,79^. Therefore, our data suggest that behavioral improvements of *Ptchd1* KO mice might correlate with altered concentrations of KYNA in the brain.

One limitation of our study is that we did not obtain clear data concerning the relationship between the changes of KP metabolite levels and locomotor activity levels. Agydelo *et al*. reported that exercise increased KATs expression in skeletal muscle, reduced plasma KYN levels, and increased KYNA levels^80^, therefore we cannot dismiss the possibility that changes in serum concentrations of KP metabolites are simply related to the hyperactivity of *Ptchd1* KO mice. However, we found that treating *Ptchd1* KO mice with ATX significantly decreased KYN concentrations and reversed certain behavioral abnormalities. Therefore, there might be an association between the pathological mechanism of ADHD/ASD and imbalance of KP metabolites in *Ptchd1* KO mice. This suggests that the KP metabolite imbalances observed in *Ptchd1* KO mice are relevant to the pathophysiological mechanisms underlying ADHD/ASD. This is important because the notion that changes in KP metabolite levels are associated with the pathological conditions of neurodevelopmental disorders, such as ADHD and ASD, is still controversial. Another limitation of our study is that we do not have enough data to illustrate the association between abnormal KP metabolite levels and the deficient *Ptchd1* gene. Therefore, further studies are needed to investigate the pathophysiological mechanism underlying abnormal KP metabolites in *Ptchd1* KO mice, an animal model of comorbid ADHD and ASD.

In conclusion, our data suggest that *Ptchd1* KO mice should be considered an animal model of ADHD and/or ASD with good predictive validity. Importantly, we found that activating KP metabolism in *Ptchd1* KO mice might change the balance of KP metabolites and increased the production of downstream metabolites, such as QUIN, in the brain. The findings strongly suggest that neuroactive compounds of the KP may modify neuronal function and/or neurodevelopment in *Ptchd1* KO mice, and further studies are required to investigate whether KP metabolites can be used as potential peripheral biomarkers for neurodevelopmental disorders. Our findings provide the basis for further examinations of KP impairments in *Ptchd1* KO mice during the prenatal stage to clarify the role of KP impairments in pathological processes. Future studies employing other genetically modified mouse strains designed to ADHD and/or ASD model are needed to evaluate whether changes in KP metabolites can be used as a clinical biomarker for improving specificity when diagnosing neurodevelopmental disorders.

## Materials and Methods

### Animals

*Ptchd1* KO mice were generated by homologous recombination in C57BL/6N embryonic stem cells and implanted in ICR blastocysts using standard procedures. Chimeric mice were crossed to female C57BL/6J mice (SLC, Japan). Germline transmission was determined by genomic PCR using three primer sets: F3 × R5, for the wild-type allele (600 bp); F4 × R5 for the Neo cassette (870 bp); and F5 × R5 as the common primer set for the WT (4.1 kbp) and mutant (2.3 kbp) alleles (Table 2, Supplemental Fig. S1A). KO mice were backcrossed to C57BL/6J mice for more than eight generations. For all experiments, *Ptchd1*^+/−^ (heterozygous) females were bred with *Ptchd1*^Y/+^ (WT) males in the C57BL/6J background. Three to five animals per cage were housed at a 12:12 h light/dark cycle (lights on at 7:00 a.m.) in a temperature- and humidity-controlled animal facility. Mice had free access to food and water. Offspring at 8 to 11 weeks of age were used for behavioral tests. Immediately after the end of behavioral tests, mice were euthanized under anesthesia for collecting the blood and brains. The protocols for all animal experiments were approved by the Committee and Animal Care at the Doshisha University, and all experiments were performed in accordance with approved guidelines and regulations.

**Table 2:** Primer sequences for genomic PCR.

Name	Sequence	
F3	WT type allele	5′-AGG TGA GAC CAG GGC GGG TTC TGG C-3′
F4	Neo cassette	5′-TCA TCG ACT GTG GCC GGC TGG GTG T-3′
F5	Common	5′-AGG ACA GCT GGG CAT TGC AGT GTC T-3′
R5	Common	5′-ATC AGG AAC CGC ACT TTG GTT GGG G-3′

WT, wild type.

### Drug administration

ATX was purchased from Tokyo Chemical Industry Co., Ltd. (Tokyo, Japan) and was suspended in 0.9% NaCl solution (saline). WT and *Ptchd1* KO mice were subcutaneously injected saline or 3.0 mg/kg ATX, acutely or chronically. For acute treatment, behavioral tests were performed 30 min after the injection. For chronic treatment, mice were injected once a day for 2 weeks before and during behavioral tests. Behavioral tests were performed 30 min after the injection. The dose of ATX was chosen based on previous studies^60,81,82^.

### Behavioral tests

All behavioral tests were performed between 9:00 a.m. and 6:00 p.m. Male offspring at the age of 8 to 11 weeks were used. To reduce the influence of previous experiments, the sequences of behavioral testing was in the order of stress degrees, from low to high. Behavioral experiments were performed in a sound-attenuated and air-regulated experimental room, to which mice were habituated for more than 1 hour before each experiment.

#### Open-field test (OFT)

The OFT was performed according to the method outlined in previous studies with minor modifications^42^. To measure locomotor activity in a novel environment, each mouse was placed in a transparent acrylic cage with a gray frosted Plexiglas floor (40 × 40 × 30 cm), and the locomotion behaviors and number of line crossing were measured every 1 min for 10 min using the Any-maze video-tracking system (Stoelting Co., Wood Dale, IL). The arena was cleaned between testing sessions with 70% alcohol. To assess the process of habituation to the novelty of the arena, mice were exposed to the apparatus for 10 min per day in 2 consecutive days. Habituation behavior was evaluated as the percentage of total distance traveled on the second day to that traveled on the first day. Although habituation in the OFT upon repeated exposures depends on the inbred mouse strain, inbred C57BL/6J male mice normally show habituation and decreased activity levels^83^.

#### Cliff avoidance test

For this test, we used a round platform and an inverted glass breaker with a diameter of 13 cm and a height of 20 cm, two times more than the mouse body length. The test was monitored by video-recorded cameras for 5 min until the mouse jumped off the platform^84,85^. The trial was terminated if mice did not jump off within 5 min. The average jumping latency and cumulative jumping events were determined by the Kaplan-Meier method and used as indices of impulsivity.

#### Y-maze test

Spontaneous alternation behavior in a Y-maze was used as an index of short-term memory. The test was performed according to the method outline in previous reports^42^. The Y-maze apparatus was made of a gray frosted Plexiglas, with each arm measuring 40 × 10 × 12 cm (L × W × H), tapering to 3-cm wide at the bottom. The arms converged to a triangular center (4 cm per side). Each mouse was placed at the end of one arm and allowed to move freely throughout the maze during an 8-min session. The series of arm entries was recorded visually. Spontaneous alternation behavior was defined as the consecutive entry into all three arms (A, B, and C) in triplet sets (i.e., ABC, ACB, BAC, BCA, CAB, and CBA). Alternation behavior was calculated as the ratio of actual to possible alternations (defined as the total number of arm entries − 2) × 100, and was presented as a percentage, as described previously.

#### Novel object recognition test (NORT)

The NORT was performed in accordance with the method outline in previous studies with minor modifications^86,87^. The test procedure consisted of three sessions: habituation, training, and retention. The habituation session consisted of a 10-min exploration time in an acrylic box (30 × 30 × 35 cm) without any objects present for 2 days. During the training session, two objects were placed in the back corner of the box. The objects included a wooden square pyramid, a golf ball, and a metal dry cell with a cylinder shape, which were different in shape, color, and material but were similar in size. A mouse was placed midway towards the front of the box, and the total time spent exploring the object was recorded for 10 min. A mouse was considered to be exploring the object when its head was facing the object or when it was touching or sniffing the object. During the retention session, the mouse was placed back into the same box 24 h after the training session, but one familiar object used during training session was replaced with a novel object. The mouse was then allowed to explore freely for 5 min, and the time spent exploring each object was recorded. Throughout the experiments, the objects were used in a counterbalanced manner in terms of their physical complexity and emotional neutrality. The discrimination index, i.e., the ratio of time spent exploring the novel object (retention session) over the total time spent exploring both objects, was used to measure cognitive function.

#### Three-chamber social approach test

This test is known as Crawley’s sociability and preference for social novelty protocol and has been successfully employed to study social affiliation and memory. The test was performed according to the method outline in previous studies with minor modifications^88,89^. Experimental mice were habituated in a three-chamber arena for 10 min/day for 2 days before the test onset. Age- and size-matched C57BL/6J male target subjects (‘stranger 1’ and ‘stranger 2’) were habituated by placing them inside wire cages for 30 min before testing. The social test apparatus consisted of an acrylic box (57 × 45 × 30 cm) with partitions dividing the box into three chambers. The wire cages used to contain the stranger male mice were cylindrical and 11-cm high, with a bottom diameter of 10.5 cm and bars spaced 1-cm apart (Galaxy Cup, Spectrum Diversified Designs). For the sociability test, the test animal was introduced to the middle and left chamber to habituate for 10 min. Following this period, the middle chamber doors were opened, and the test mouse was allowed to freely explore all three chambers for additional 10 min. The test mouse was then returned to the middle chamber; subsequently, an unfamiliar mouse (stranger 1) was introduced into the wire cage in one of the side-chambers, while the other wire cage was empty (Empty) on the other side-chamber. The dividers were then raised, and the test animal was allowed to freely explore all three chambers over a 10-min session. Following this session (session 1), the mouse was returned to the middle chamber, and a novel stranger mouse (stranger 2) was put in the previously empty wire cage; the test animal was allowed to explore for a 10-min session again (session 2). The time spent by the mouse (nose-point) in close proximity (~2 cm) to the wire cages was measured as interaction time (i.e. sniffing time) to estimate the approach behavior. Sessions were video-recorded, and the object exploration time and total distance moved were analyzed using the Any-maze tracking system. Arenas and contents were thoroughly cleaned between testing sessions. Multiple social targets from different home cages were used for testing to prevent potential odorant confounds from target home cages.

#### Five-trial social recognition memory test

This test assesses cognition and sociability, namely the ability to recognize novel versus familiar animals. This test was performed according to the method outline in previous studies with minor modifications^90^. Experiment mice were habituated in the test arena for 10 min/day for 2 days before testing. Age- and size-matched C57BL/6J male stimulus mice were used and habituated to a cylinder holder made of transparent acryl (30-cm high, 10.5-cm diameter, with 16 holes of 0.5-cm diameter) for 30 min before testing. In this test, the same stimulus animal was used for both acquisition and recognition phases (intruder 1). Over the course of multiple exposures, experiment mice became habituated to intruders and no longer regarded them as interesting as they did for completely novel intruders. During testing, each experimental mouse was given four 5-min exposures, with 15–20 min of intervals, in a transparent acrylic box (60 × 60 × 30 cm). In the fifth trial, the experimental mice encountered an entirely novel intruder (intruder 2). All test trials were video-recorded using the Any-maze tracking system, and the total investigation time was subsequently analyzed. The time spent by each mouse (nose-point) in close proximity (~2 cm) to the cylinder holder was measured as interaction time (i.e. sniffing time, approach) to estimate the approach behavior.

### Measurements of KP metabolites

The serum was diluted (4:1, v/v) in 10% perchloric acid (PCA) for measurements of KP metabolites. After thorough mixing, the precipitated proteins were removed by centrifugation (7,000 g × 10 min, 4 °C), as described previously^91^. Brain tissues were weighed and homogenized (1:3, w/v) in 10% PCA. After homogenization, the precipitated proteins were removed by centrifugation (17,000 g × 15 min, 4 °C), and 50 μL of the supernatant was subjected to high-performance liquid chromatography (HPLC) analysis for measurements of KP metabolites in the serum and frontal cortex. TRP, KYN, KYNA, AA, and 3-HAA were isocratically eluted from a reverse-phase chromatography column [TSK-GEL ODS-100 V, 3-μm particle size, 4.6 mm (ID) × 150 mm (L); TOSOH Co., Tokyo, Japan] with a mobile phase containing 10 mM sodium acetate and 1% acetonitrile (pH adjusted to 4.5 with acetic acid) at a flow rate of 0.9 mL/min. TRP and KYN were detected using an ultraviolet and visible spectrophotometric detector (UV detector, SPD-20A, Shimadzu Co., Kyoto, Japan) (UV wavelength for TRP: 280 nm, for KYN: 365 nm). KYNA, AA, and 3-HAA were detected by a fluorescence spectrometric detector (RF-10Axs, Shimadzu Co., Kyoto, Japan) under the following conditions: for KYNA, the excitation (Ex) wavelength was 334 nm, and the emission (Em) wavelength was 380 nm; for AA and 3-HAA, the Ex wavelength was 320 nm, and the Em wavelength was 420 nm. 3-HK was measured by HPLC with an electrochemical detector (Eicom ECD-300; oxidation potential: + 0.55 V) and a chromatographic column [EICOMPAK SC-50DS, 3-μm particle size, 3.0 mm (ID) × 150 mm (L); Eicom Co., Kyoto, Japan] with a mobile phase of 0.59% (v/v) phosphoric acid, 0.27 mM EDTA, 8.9 mM sodium heptane sulfonic acid, 0.9% (v/v) trimethylamine, and 1.5% (v/v) acetonitrile at a flow rate of 0.5 ml/min, as described previously^42^.

### Statistics

All data are expressed as mean ± standard error of the mean (SEM) for each group. The unpaired Student’s *t*-test was used to compare two sets of data. In case of more than three groups, two-way ANOVA was used, followed by the Bonferroni’s multiple comparison test. In the cliff avoidance test, the data for cumulative jumping events were compared by the log-rank test with Bonferroni correction. Statistical analysis was performed, and graphs were generated using GraphPad Prism version 5.0 (GraphPad Software, San Diego, USA). *P* values of less than 0.05 were considered statically significant.

## Supplementary information

Supplemental information
