# Author Correction: Altered kynurenine pathway metabolites in a mouse model of human attention-deficit hyperactivity/autism spectrum disorders: A potential new biological diagnostic marker

**DOI:** 10.1038/s41598-020-60585-3

**Published:** 2020-02-28

**Authors:** Yuki Murakami, Yukio Imamura, Kuniaki Saito, Daisuke Sakai, Jun Motoyama

**Affiliations:** 1 Organization for Research Initiatives and Development, Doshisha University, Kyoto, 610-0394 Japan; 2 Department of Hygiene and Public Health, Kansai Medical University, Hirakata, 573-1010 Osaka Japan; 3 Department of Traumatology and Acute Critical Medicine, Osaka University Graduate School of Medicine, Suita, 565-0871 Osaka Japan; 4 Department of Disease Control and Prevention, Fujita Health University Graduate School of Health Sciences, Toyoake, 470-1192 Japan; 5 Division of General Education, Biology, Kanazawa Medical University, Kanazawa, 920-0293 Japan; 6 Laboratory of Development Neurobiology, Graduate School of Brain Science, Doshisha University, Kyoto, 610-0394 Japan

Correction to: *Scientific Reports* 10.1038/s41598-019-49781-y, published online 12 September 2019

The original version of this Article contained a typographical error in the spelling of the author Jun Motoyama, which was incorrectly given as Jun Motyama. This has now been corrected in the PDF and HTML versions of the Article.

