# Supplementary material for: Altered kynurenine pathway metabolites in a mouse model of human attention-deficit hyperactivity/autism spectrum disorders: A potential new biological diagnostic marker
Source: Sci Rep. 2019 Sep 12;9:13182. doi: 10.1038/s41598-019-49781-y (PMC6742629; doi:10.1038/s41598-019-49781-y)

Supplemental information

The manuscript title: Altered kynurenine pathway metabolites in a mouse model of human attention-deficit hyperactivity/autism spectrum disorders: a potential for new biological diagnostic marker

Author list: Yuki Murakami[1, 2*], Yukio Imamura[1, 3], Kuniaki Saito[4], Daisuke Sakai[1, 5], Jun Motoyama[6]

[1] Organization for Research Initiatives and Development, Doshisha University, Kyoto 610-0394, Japan.

[2] Department of Hygiene and Public Health, Kansai Medical University, Osaka 573-1010, Japan

[3] Department of Traumatology and Acute Critical Medicine, Osaka University Graduate School of Medicine, Osaka 565-0871, Japan

[4] Department of Disease Control and Prevention, Fujita Health University Graduate School of Health Sciences, Aichi 470-1192, Japan.

[5] Division of General Education, Biology, Kanazawa Medical University, Ishikawa 920-0293, Japan

[6] Laboratory of Development Neurobiology, Graduate School of Brain Science, Doshisha University, Kyoto 610-0394, Japan.

Short title: Kynurenine pathway metabolites as diagnostic biomarkers for ADHD/ ASD

* Corresponding author: Yuki Murakami, Ph.D.

Organization for Research Initiatives and Development, Doshisha University

HC301 1-3 Tataramiyakodani Kyotanabe City, Kyoto 610-0394, Japan

Phone: +81-774-65-6060
Fax: +81-774-73-1903
Email: ymurakam@mail.doshisha.ac.jp
Present address: Department of Hygiene and Public Health, Kansai Medical University
2-5-1 Shinmachi, Hirakata City, Osaka, 573-1010, Japan
Phone: +81-72-804-2402
Fax: +81-72-804-2409
Email: murakamy@hirakata.kmu.ac.jp

Supplemental Methods

Drug Administration

Methylphenidate (MPH) was purchased from Sigma-Aldrich/Merck Millipore (Darmstadt, Germany) and was suspended in 0.9% NaCl solution (saline). Wild-type (WT) and *Ptchd1* knockout (KO) mice were subcutaneously injected saline or 0.5 mg/kg MPH. Behavioral tests were performed 30 min after the injection. The dose of MPH, which was chosen based on previous studies [60,80,81], did not affect any behavioral tests in WT mice.

Supplemental Figure Legends

Fig. S1 *Ptchd1* knockout (KO) mouse generation and validation. (A) A schematic diagram describes the strategy to create *Ptchd1* KO mice. (B) Genomic PCR confirms deletion of exon 1 from the genome of male KO mice. Het, heterozygous; WT, wild-type.

Fig. S2 No anxiety-like and depression-like behaviors are found in *Ptchd1* knockout (KO) mice. (A) Anxiety-related behavior was measured using of the open filed. A decrease in time spent in the center of the open field, which is an indicator of increased anxiety-like behavior, was not observed in *Ptchd1* KO mice compared with wild-type (WT) mice (Student's *t*-test, t = 0.394, $p > 0.05$ versus WT). (B) Immobility time in the forced-swim test (FST) in WT and *Ptchd1* KO mice. *Ptchd1* KO mice showed significantly reduced immobility time in the FST compared with WT mice (Student's *t*-test, t = 4.206, $p < 0.001$ versus WT).

Fig. S3 Social behaviors of *Ptchd1* knockout (KO) mice following acute and chronic treatment with atomoxetine (ATX). (A, B) Acute administration of ATX. Behavioral tests were performed 30 min after the injection. (C, D) Chronic administration of ATX. Mice (6 weeks of age) were injected ATX once per day, and behavioral tests were performed 30 min after the last injection. (A, C) Three-chamber social approach test. Statistical analysis was conducted using two-way analysis of variance (ANOVA) with Bonferroni's multiple comparison test: *, $p < 0.05$, **, $p < 0.01$, ***, $p < 0.001$ versus empty cage or stranger 1 (familiar mouse); $^{\#}$ $p < 0.05$, $^{\#\#}$, $p < 0.01$ versus WT+ATX with stranger 2 (unfamiliar mouse). (B, D) Five-trial social recognition memory test. Statistical analysis was performed using two-way ANOVA with Bonferroni's multiple comparison test: *, $p < 0.05$, **, $p < 0.01$ versus WT+sal. Each column represents mean ± standard error of the mean (SEM; n = 10-24). Sal, saline; Empty, empty cage; St1, stranger 1; St2, stranger 2.

Fig. S4 Acute treatment with methylphenidate (MPH) does not improve behavioral

abnormalities in *Ptchd1* knockout (KO) mice. Wild type (WT) and *Ptchd1* KO mice at the age of 8 weeks were administrated MPH at a dose of 0.5 mg/kg 30 min before the behavioral tests. (A) Total distance traveled in a novel environment on the first day [two-way analysis of variance (ANOVA) with Bonferroni's multiple comparison test: **, $p < 0.01$ versus WT+sal; $^{###}$, $p < 0.001$ versus WT+MPH]. (B) Percentage of total distance traveled on the second day to that on the first day, a measure of habituation to a novel environment (two-way ANOVA with Bonferroni's multiple comparison test: *, $p < 0.05$ versus WT+sal). (C) Impulsivity in the cliff avoidance test. The cumulative curve of the jumping events was generated by the Kaplan-Meier method (log-rank test with Bonferroni's correction: *, $p < 0.05$ and ***, $p < 0.001$ versus WT+sal; $^{\#}$, $p < 0.05$ and $^{##}$, $p < 0.01$ versus WT+MPH). The average jumping latency is shown (two-way ANOVA with Bonferroni's multiple comparison test: ***, $p < 0.001$ versus WT+sal; $^{##}$, $p < 0.01$ versus WT+MPH). (D) Spontaneous activity and working memory in the Y-maze test. Total arm entries (two-way ANOVA with Bonferroni's multiple comparison test: ***, $p < 0.001$ versus WT+sal; $^{##}$, $p < 0.01$ and $^{###}$, $p < 0.001$ versus WT+MPH) and alternation behavior (two-way ANOVA with Bonferroni's multiple comparison test: **, $p < 0.01$ versus WT+sal; $^{##}$, $p < 0.01$ and $^{###}$, $p < 0.001$ versus WT+MPH) were measured during an 8-min session. (E) Objective recognition memory was measured in the novel object-based recognition test. Memory retention session was assessed 24 h after the training session, and the discrimination index was calculated as shown in Methods (two-way ANOVA with Bonferroni's multiple comparison test: ***, $p < 0.001$ versus WT+sal; $^{###}$, $p < 0.001$ versus WT+MPH). (F) Three-chamber social approach test. Statistical analysis was performed using two-way ANOVA with Bonferroni's multiple comparison test: *, $p < 0.05$ and ***, $p < 0.001$ versus empty cage or stranger 1 (familiar

mouse). (G) Five-trial social recognition memory test. Statistical analysis was performed using two-way ANOVA with Bonferroni's multiple comparison test: *, $p < 0.05$, **, $p < 0.01$ and ***, $p < 0.001$ versus WT+sal; $^{\$}$, $p < 0.05$ versus KO+sal. Each column represents mean ± standard error of the mean (SEM; n = 6-10). Sal, saline; Empty, empty cage; St1, stranger 1; St2, stranger 2.

Fig. S5 Schematic diagram of the kynurenine pathway (KP). Most dietary L-tryptophan (L-TRP) is used for protein synthesis. Indoleamine 2,3-dioxygenase 1 (IDO1) or L-tryptophan 2,3-dioxygenase (TDO) catabolizes L-TRP to form N-formyl-L-kynurenine, which is converted to L-kynurenine (L-KYN) by formamidase. Less than 1% of L-TRP is converted to serotonin (5-HT) in the brain. L-KYN is further metabolized to anthranilic acid (AA) by kynureninase (KYNU), to kynurenic acid (KNYA) by kynurenine aminotransferases (KATs), and to 3-hydroxykynurenine (3-HK) by kynurenine 3-monopxygenase (KMO), which is metabolized to 3-hydroxyanthranilic acid (3-HAA) by 3-hydroxyanthranilate 3, 4-dioxygnase (3-HAAO). 3-HAA is further metabolized to quinolinic acid (QUIN). Some 3-HAA is converted to picolinic acid (PA) by 2-amino-muconic semialdehyde (ACMSD).

Fig. S6 Comparison of the concentrations of kynurenine pathway (KP) metabolites in the serum between *Ptchd1* knockout (KO) and wild-type (WT) mice at different ages. KP metabolite concentrations were determined in the serum in mice at different ages (6, 8, 11, 12, and 14 weeks). Open and closed bars correspond to WT and *Ptchd1* KO mice, respectively. Each column represents mean ± standard error of the mean (SEM; n = 6-17). Statistical analysis was performed using Student's *t*-test at each age: *, $p < 0.05$,

**, $p < 0.01$ versus WT mice.

Supplemental Fig. S1

(A)

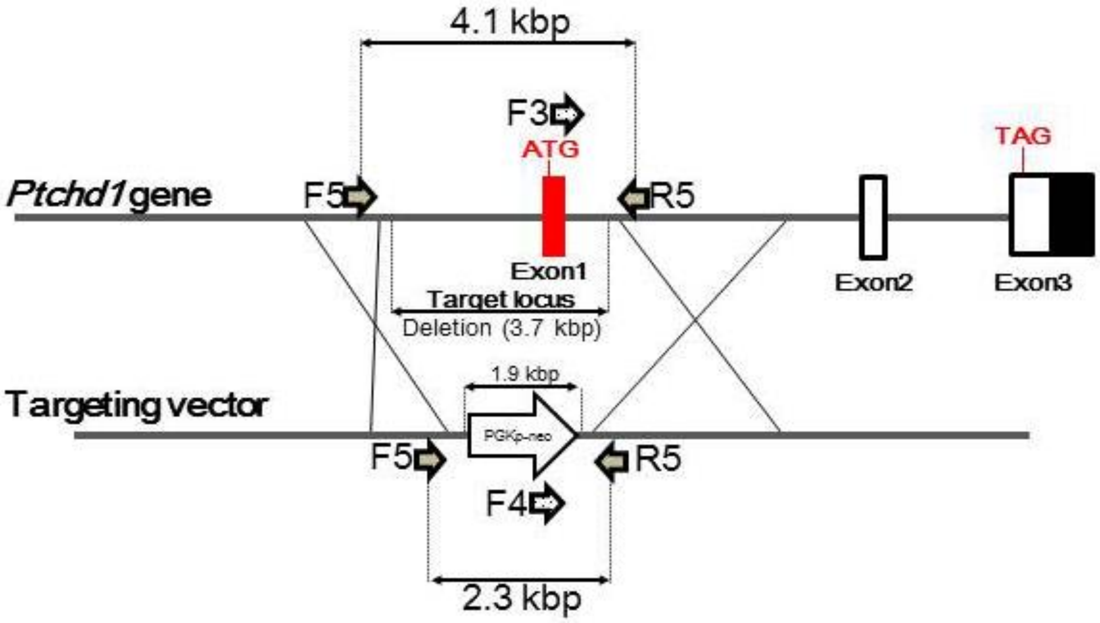

(B)

Genomic PCR (F5 x R5)

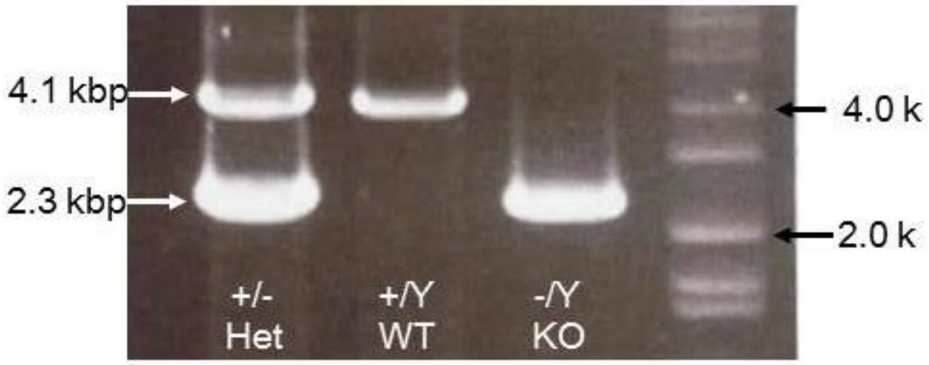

Supplemental Fig. S2

(A) Anxiety like behavior

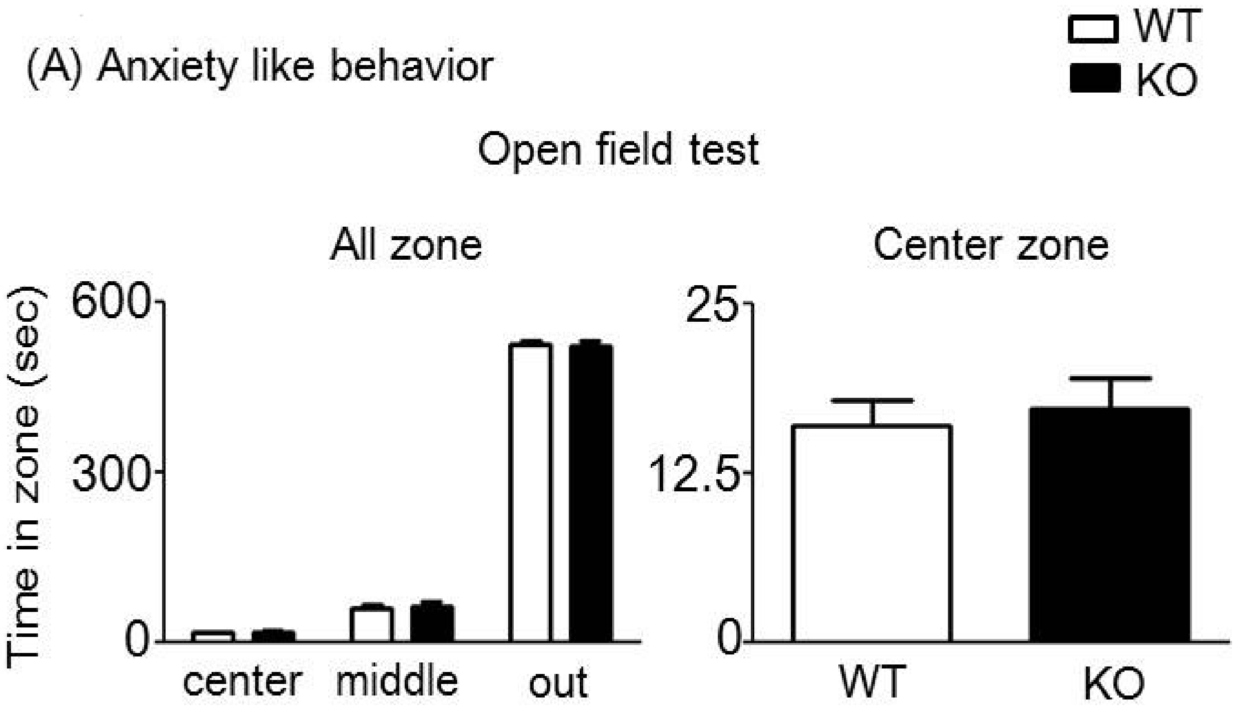

(B) depression like behavior

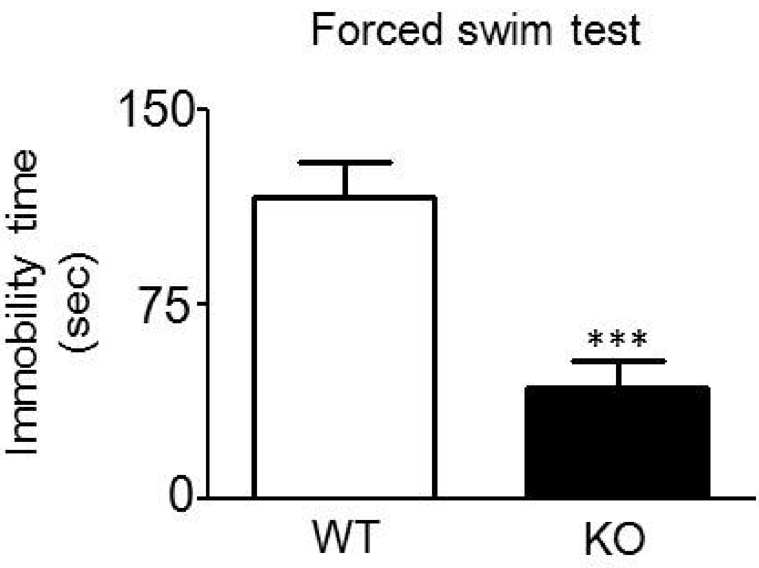

Supplemental Fig. S3A, B

Acute administration

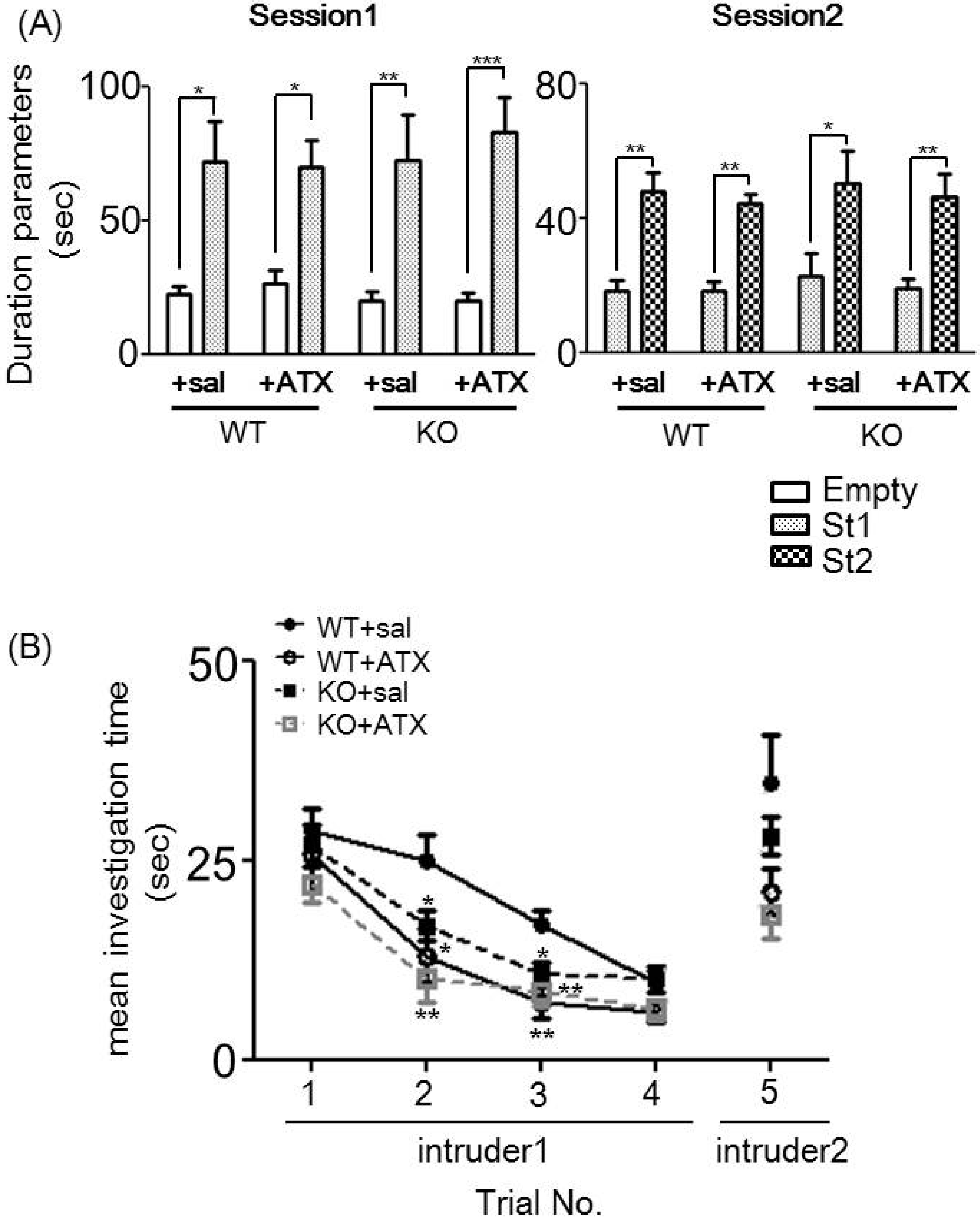

Supplemental Fig. S3C, D

Chronic administration

(C)

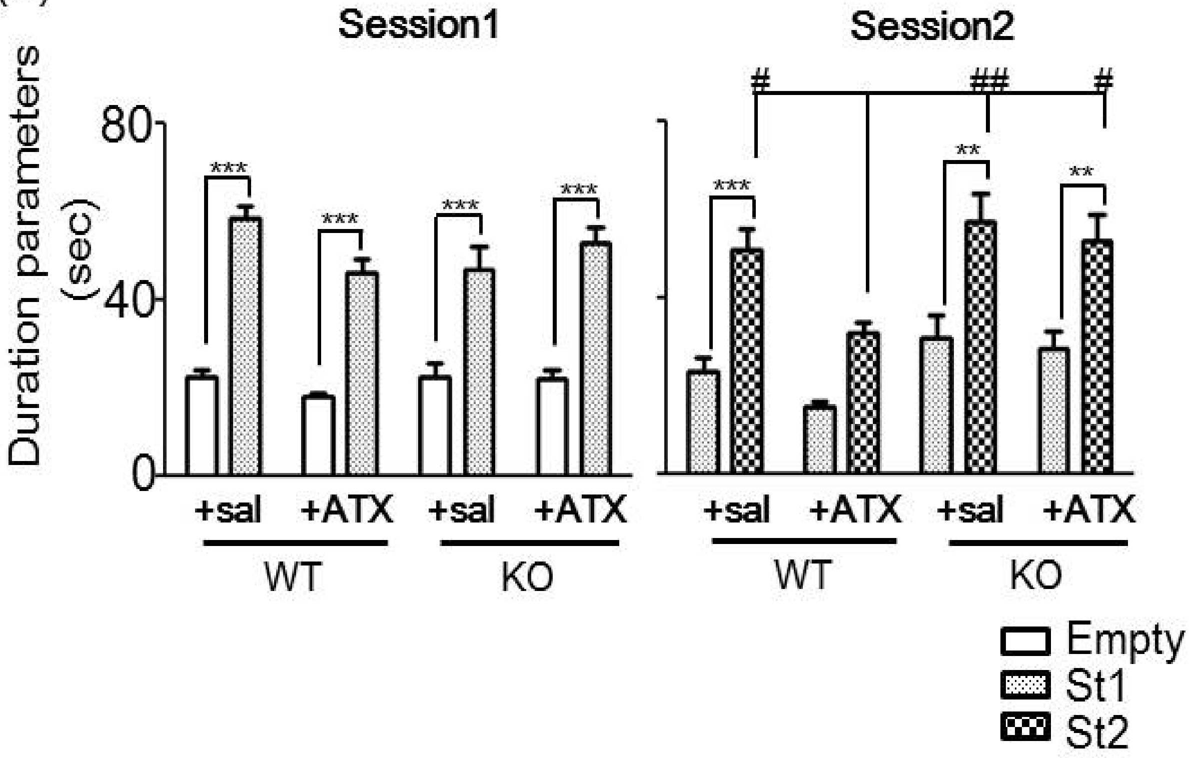

(D)

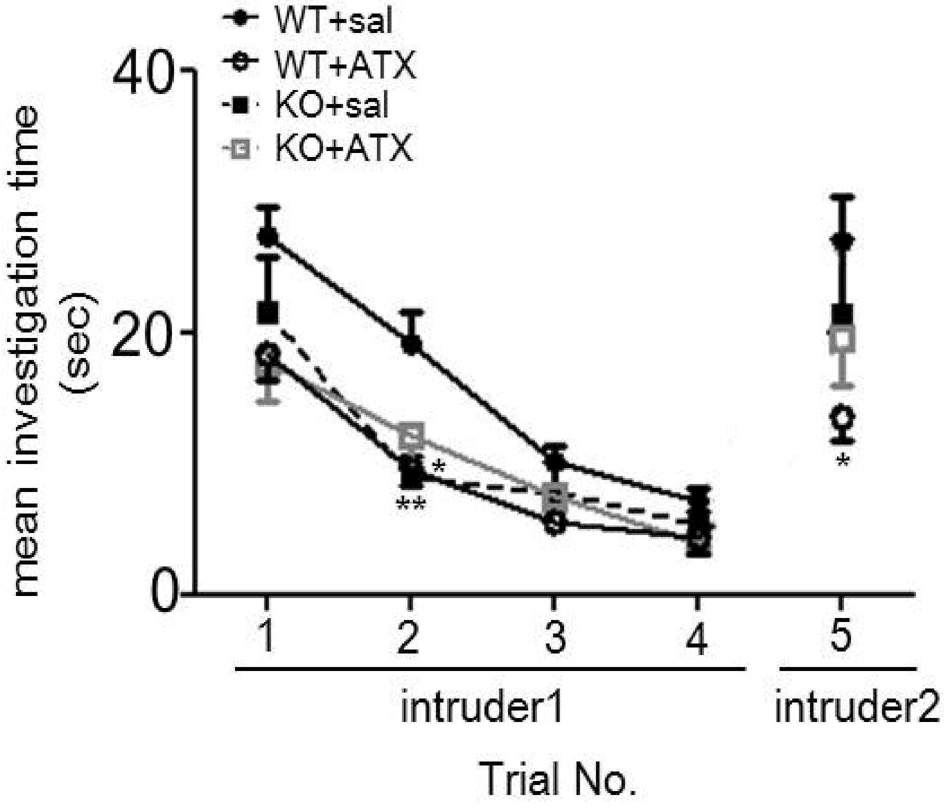

Supplemental Fig. S4A, B, C

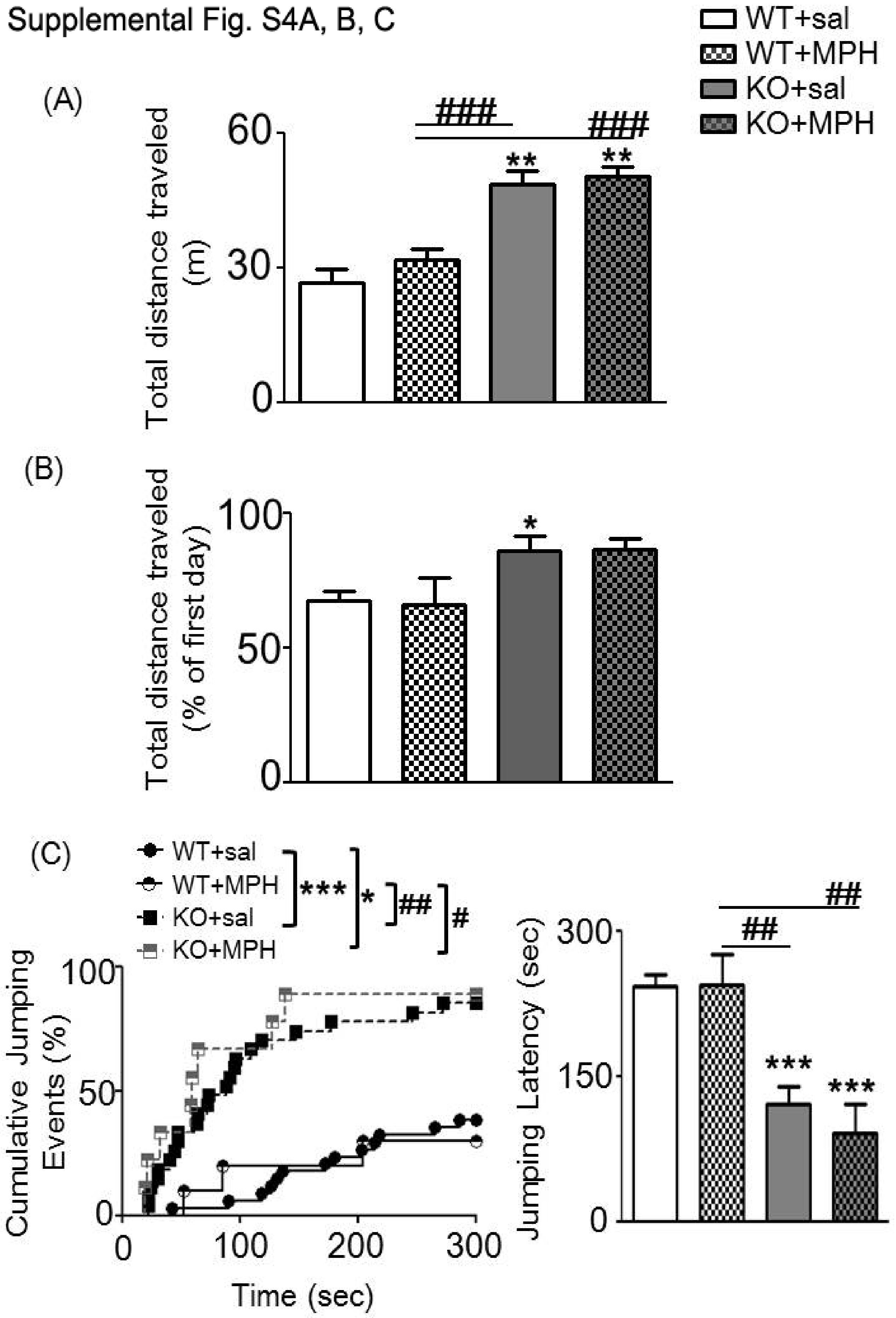

Supplemental Fig. S4D, E

(D)

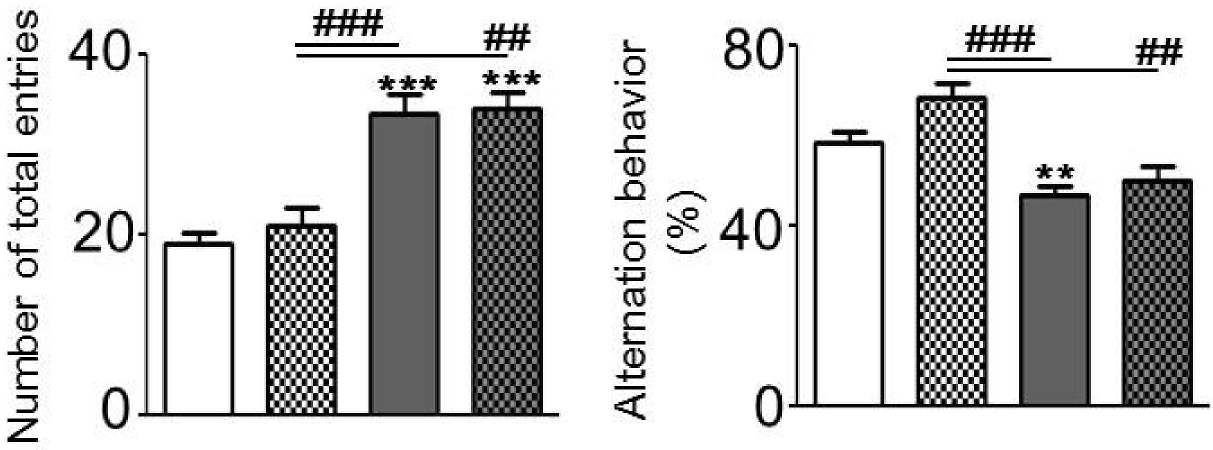

(E)

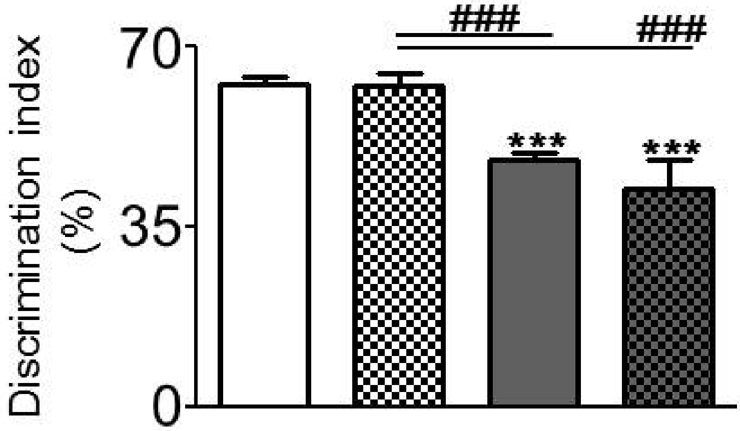

Supplemental Fig. S4F, G

(F)

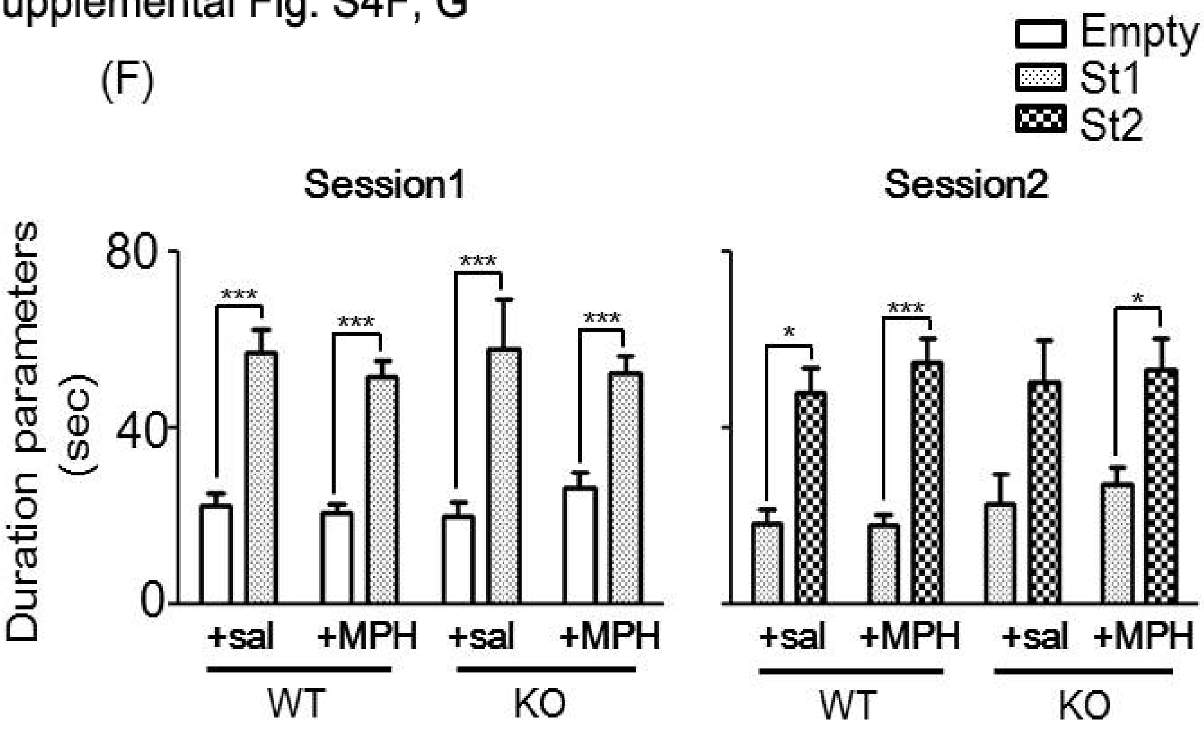

(G)

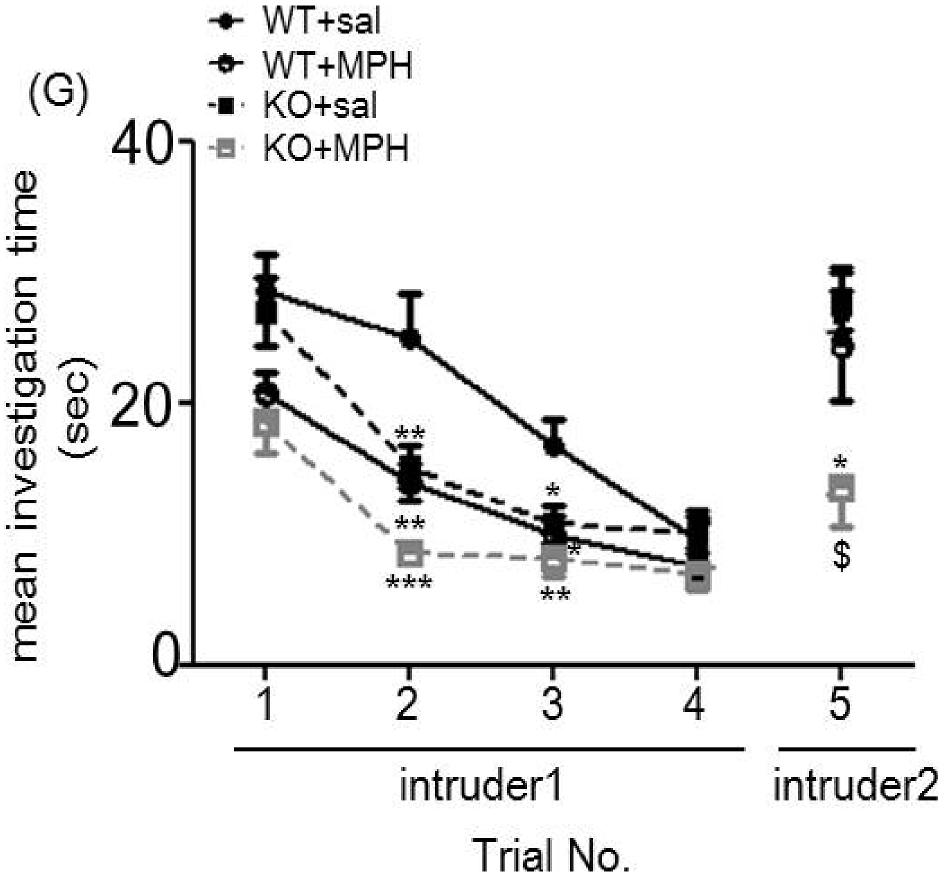

Supplemental Fig. S5

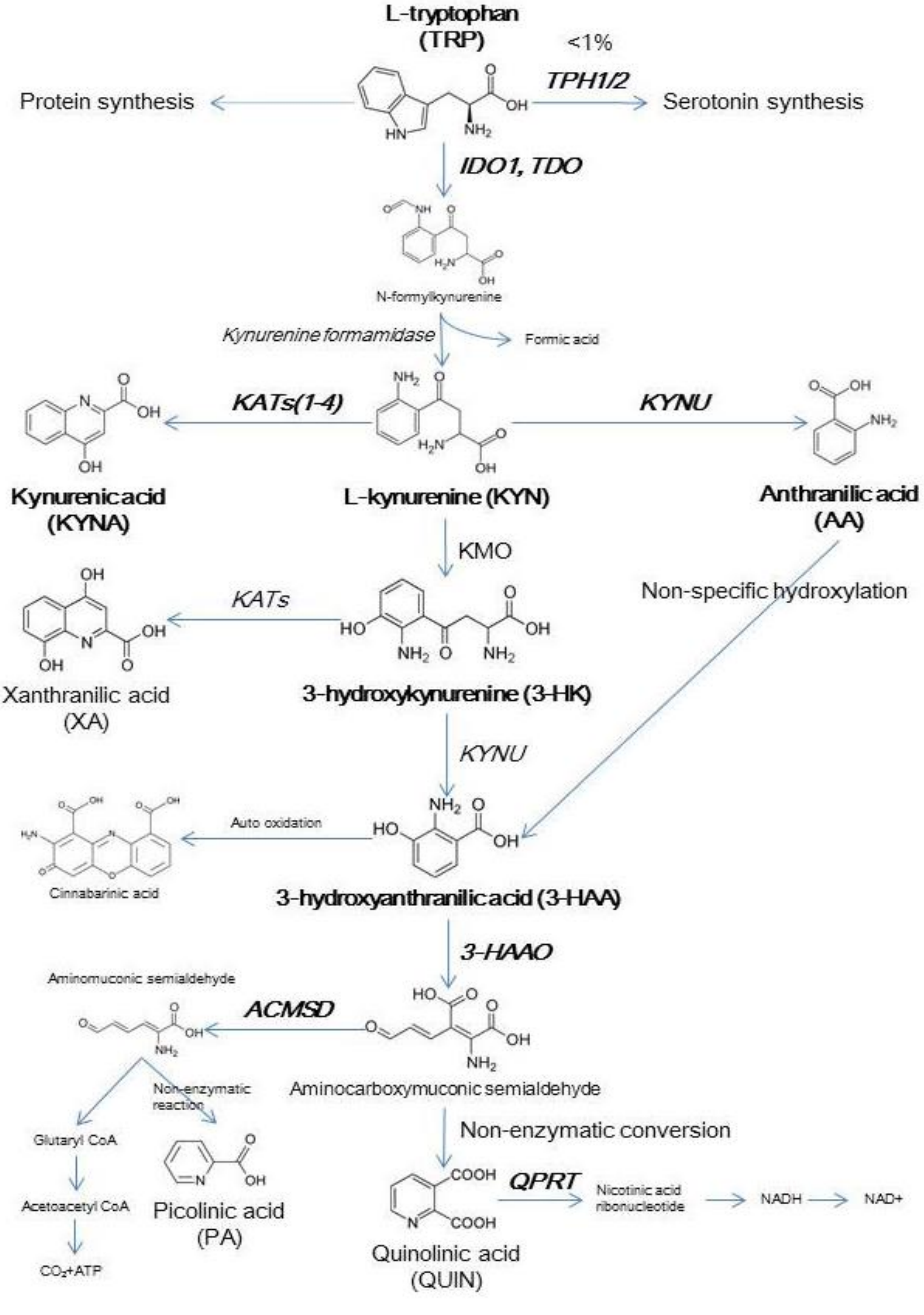

Supplemental Fig. S6

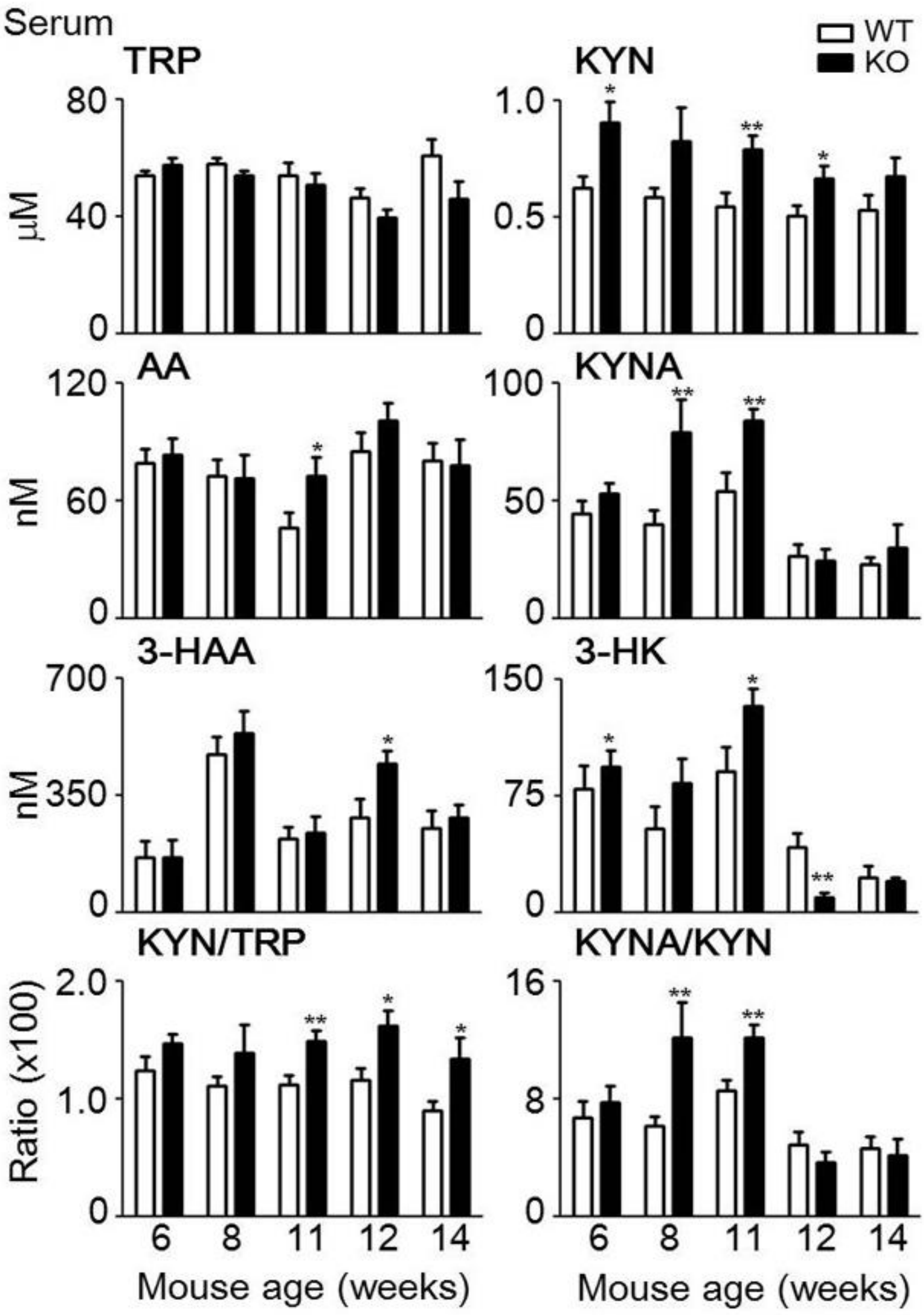

Raw data

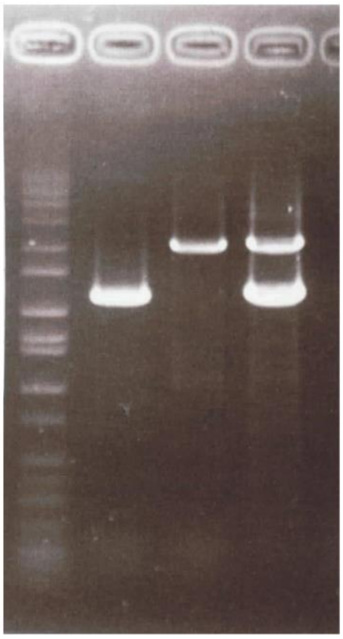

Supplement: Supplementary file 1 — Supplemental information [file 41598_2019_49781_MOESM1_ESM.pdf]
